# Coordination of Single-cell and Bulk RNA Sequencing to Construct a Cuproptosis-related Gene Prognostic Index for Endometrial Cancer Prognosis, Immune Microenvironment Infiltration, and Immunotherapy Treatment Options

**DOI:** 10.7150/jca.86325

**Published:** 2023-09-18

**Authors:** Xiaoao Pang, Feifei Li, Miao Lu, Liancheng Zhu

**Affiliations:** 1 Department of Obstetrics and Gynecology, Shengjing Hospital of China Medical University, Shenyang, Liaoning, China.; 2 Department of Gynecology, Shandong Provincial Hospital Affiliated to Shandong First Medical University, Jinan, China.

**Keywords:** Cuproptosis, endometrial cancer, prognosis, immune microenvironment, immune checkpoint inhibitor (ICI).

## Abstract

**Background**: This study aims to identify molecular subtypes and develop a cuproptosis-related gene prognostic index (CRGPI) for endometrial cancer (EC), in addition to outlining the immune features and the efficacy of immune checkpoint inhibitor (ICI) therapy in CRGPI-defined groups of EC.

**Methods**: Between malignant and normal cells distinguished from single-cell RNA sequencing data GSE154763 dataset, the difference in KEGG pathways and cuproptosis-related gene (CRG) scores was intensively investigated. On the basis of TCGA dataset (n=492), CRGs were used to identify two distinct molecular subtypes. Using the Cox regression method, a CRGPI was constructed and externally validated with the IMvigor210 dataset (n=348) and GSE78220. Then, the molecular and immune characteristics and the efficacy of ICI therapy in subgroups defined by CRGPI were investigated.

**Results**: Compared to normal cells, the expression of the TCA cycle and CRGs genes was significantly higher in malignant cells. The CRGPI was established on the premise of ATF5, FBXO46, P2RX4, SMARCD3, DAPK3, and C1orf53. Comprehensive results demonstrated a correlation between a low CRGPI score and better overall survival, younger age, early stage, immune cells and functions activation, high tumor mutation burden and high microsatellite instability, as well as better benefit from ICI therapy, and its significance for forecasting immunotherapeutic effects was verified as well (IMvigor210 cohort: HR, 1.358; 95% CI, 1.047, 1.761; p=0.02; GSE78220 cohort: HR, HR = 3.857, 95% CI, 1.009, 14.74; p=0.034). CRGPI anticipated the immunotherapy medication.

**Conclusions**: CRGPI is a prospective biomarker to estimate the prognosis, immune and molecular characteristics, and treatment benefit of immunotherapy in EC.

## Introduction

Copper is an essential nutrient with oxidation-reduction (redox) properties that allow it to promote copper-dependent cell growth and proliferation (cuproplasia) and mitochondrial-dependent cytotoxicity (cuproptosis) when it exceeds a certain threshold 1. Copper directly binds to lipoylated components of the tricarboxylic acid cycle, resulting in the accumulation of lipoylated protein and subsequent deletion of iron-sulfur cluster protein, which ultimately leads to proteotoxic stress and cell death, as confirmed by numerous research studies 2. The specific process of excessive copper-induced cell death was not clarified until March 2022, when Tsvetkov et al. demonstrated that copper death was distinct from the established mechanism of cell death 3. They demonstrated that copper-induced cell death is distinct from all known regulatory cell death mechanisms, including apoptosis, ferroptosis, pyroptosis, and necroptosis. Consequently, the authors propose naming this previously unclassified mode of cell death cuproptosis 3. With a precise definition of cuproptosis, future research on cuproptosis-related regulatory factors in cancer may offer not only potential processes for the development and treatment of cancer, but also novel ideas for the classification, outcome, and prognosis of cancer's therapeutic responsiveness.

Endometrial carcinoma (EC) is the most common malignant gynecological malignancy found in the female reproductive tract 4. Globally, it is estimated that 76,000 women die each year 5. In recent years, the number of reported cases has increased, the average age of onset has decreased, and the mortality rate has risen more quickly than the incidence rate 6. Currently, there is no biomarker or signature capable of accurately predicting the survival rate of EC patients. In order to improve the overall prognosis of EC patients, it is crucial to identify the predictive and prognostic characteristics of patients at high risk.

Immune checkpoint inhibitor (ICI) therapies, such as those targeting programmed death 1 (PD1), programmed death ligand 1 (PD-L1), and CTL associated protein 4 (CTLA4), have fundamentally altered the treatment of a variety of malignancies, especially advanced cancers, in comparison to conventional therapies 7, 8. Despite the fact that some patients experience remarkable and long-lasting disease regression as a result of ICIs, the majority of patients do not benefit from these treatments, and some may experience the adverse phenomenon of hyperprogressive disease 8. Consequently, the recognition of prospective prognostic indicators associated with therapeutic benefit may make the immunotherapy of EC patients more personalized and specific, thereby raising the possibility of a highly precise treatment for EC patients.

## Materials and methods

### Data gathering and preliminary processing

Single-cell RNA sequencing (scRNA-seq) data GSE154763 was downloaded from GEO platform, which detected a total of 9 EC tissues based on the 10X Genomics. The TCGA database (https://portal.gdc.cancer.gov/) was utilized to collect EC patient transcriptome data (FPKM value), clinical information, and mutation data. In the subsequent studies, a total of 492 EC patients were included. Clinical factors included age, stage, grade, myometrial invasion percentage, overall survival time, and survival status. The RNA-seq data of 50 undifferentiated uterine sarcoma samples (GSE119041) was downloaded. The flowchart in this study is illustrated in Figure S1.

### scRNA data Processing

The processing of scRNA-seq data was described in a previous report 9. The "CellCycleScoring" algorithm was then used to predict classification of each cell as either S, G2M, or G1 phase. The "copykat" package was utilized to predict the presence of diploid (normal cells) and aneuploid (tumor cells).

### Unsupervised clustering for cuproptosis-related genes

Nineteen CRGs were identified in previous investigations 3, 10-12. The "ConsensusClusterPlus" R package 13 was used to divide all 492 EC patients into distinct molecular subgroups based on CRG expression for consensus unsupervised clustering analysis.

### Functional enrichment analysis

The variation in OS between subtypes was analyzed by Kaplan-Meier analysis. Gene set variation analysis (GSVA) was applied to the MSigDB-derived hallmark gene set (c2.cp.kegg.v7.2) to study changes in CRGs in biological processes 14.

### Evaluation of tumor microenvironment cells

The scores of tumor microenvironment (TME) cells in every EC sample was evaluated by single sample gene set enrichment analysis (ssGSEA) algorithm 15. Using the ESTIMATE method 16, immunological and stromal scores for each patient were determined. Using the CIBERSORT algorithm, the fractions of 22 human immune cell types in each EC sample were determined.

### Identification of differentially expressed genes

Using the "limma" package in R, the differentially expressed genes (DEGs) among the cuproptosis clusters were identified with |fold change|>2 and p-value <0.001. Gene ontology (GO), Kyoto Encyclopedia of Genes and Genomes (KEGG) pathway enrichment analysis, and the protein-protein interaction network were performed with Metascape 17. Gene Set Enrichment Analysis (GSEA) was employed to identify the KEGG pathways that are enriched in gene rank.

### Construction of the cuproptosis-related prognostic index

Beginning with a univariate Cox regression analysis, the prognostic DEGs were identified. Then, the patients were separated into distinct subtype groups using an unsupervised clustering algorithm (cuproptosis gene Cluster A and Cluster B). The CRGPI was then calculated using the training cohort. Briefly, the LASSO Cox regression was applied to reduce the likelihood of over-fitting based on CRG prognostic genes 18. After analyzing the change trajectory of each independent variable, we utilized 10x cross validation to establish the CRGPI. Multivariate Cox analysis was used in the training cohort to select candidate genes for establishing the prognostic CRGPI. Six genes and their training cohort-obtained correlative coefficients were used to create the CRGPI. The following equation was used to calculate the CRGPI: CRGPI = 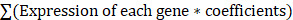. On the basis of their median values, the patients in the training cohort were separated into CRGPI-low and CRGPI-high groups, and then a Kaplan-Meier survival analysis was performed. The predictive value of the signature was assessed through the development of receiver operating characteristic (ROC) curves.

### Clinical correlation and stratification analyses of the CRGPI

Using Chi-square testing, the correlation between the CRGPI and clinical variables was investigated. On the training, testing, and total cohorts, univariate and multivariate analyses were conducted to ascertain the independence of CRGPIs from other clinicopathological variables. In addition, a stratified analysis was carried out to determine whether the CRGPI retained its predictive power across other clinical factors.

### External CRGPI verification

External validation for the prognostic significance of six genes in the model is provided by KM-plotter database (http://www.km-plotter.com/). The cohort (IMvigor210) of Mariathasan et al. 19 was also used for external validation of CRGPI.

### TMB analysis

Patients' mutation information was downloaded from the TCGA data portal. The information was stored in Mutation Annotation Format and processed by the Var-Scan software. Adoption of the "maftools" package for analyzing and demonstrating gene mutation patterns and frequencies in different groups.

### Immune status evaluation

The differential expression levels of human leukocyte antigen (HLA) family members and immune checkpoint biomarkers between the CRGPI-low and -high groups were further analyzed. Additionally, we analyzed the relationships between the two groups and microsatellite instability (MSI).

Tumor Immune Single-cell Hub (TISCH) obtained data from GEO and ArrayExpress 20 to compile its scRNA-seq atlas, a single-cell RNA-seq resource concentrating on TME. TISCH2 (http://tisch.comp-genomics.org) has 190 datasets and 6,297,320 cells from tumor patients and healthy donors 21, enabling the investigation of TME across various cancer types. Using TISCH2 datasets, we discovered the CRGPI gene heterogeneity among immune cells in endometrial cancer at the single-cell level.

### Forecasting immunotherapy response

Immunotherapy response was first evaluated by immunophenoscore (IPS) analysis 22. The Cancer Immunome Atlas (TCIA) provided the IPSs of EC patients (https://tcia.at/home). Time-dependent ROC curve analyses were performed to obtain the AUC and compared the prognostic value of CRGPI, TMB, MSI, and TIS using the timeROC package in R. The TIS score was calculated as the mean log_2_-scale normalized expression of the 18 signature genes 23.

In addition, three transcriptomic data sets with clinical data from patients with metastatic urothelial cancer treated with an anti-PD-L1 agent (IMvigor210 cohort 19), patients with metastatic melanoma treated with anti-PD-1 pembrolizumab (GSE78220 24), patients with advanced clear cell renal cell carcinoma treated with anti-PD-1 Nivolumab and anti-mTOR inhibitor everolimus (CheckMate025 25) were downloaded and analyzed to evaluate response of immunotherapy and determine the predictive value of the CRGPI 26.

### Assessment of chemotherapeutic and molecular drugs' sensitivity

In order to predict the response to chemotherapy and molecular drugs, the "pRRophetic" package employed to compute the CRGPI 27; the half-maximal inhibitory concentration (IC50) was calculated between the low and high groups for 251 common chemotherapeutic agents.

### Statistical analysis

We conducted statistical analyses using R (version 4.2.0) and RStudio (version 2022.02.2 Build 485 for macOS). The Mann-Whitney U test was used to compare differences between two groups. For the analysis of differences between more than two groups, the Kruskal-Wallis test was utilized. P < 0.05 was regarded statistically significant in all two-tailed tests, unless otherwise specified.

## Results

### scRNA-seq data quality control, normalization, and bioinformatics analysis

Due to lack of the raw data in GSE154763, we only obtained the processed data. A total of 8808 cells were obtained by filtering the single-cell data so that each gene was expressed in at least 3 cells and each cell expressed at least 300 genes. To reduce the bias caused by too large differences in sample sizes, we kept only the GSM datasets with a cell count > 500 in this study. Table 1 presents the cell count data for each sample before and after filtration. As shown in Figure S2A, there was a significant correlation (R = 0.82) between the number of UMI and mRNA. Figures S2B and C illustrate the violin before and after quality assurance. The available dimensions were estimated using principal component analysis (PCA), and the results did not disclose any significant differences between EC cells. Forty of the most distinctive primary components were chosen for additional examination (Figure S2D).

**Figure 1:**
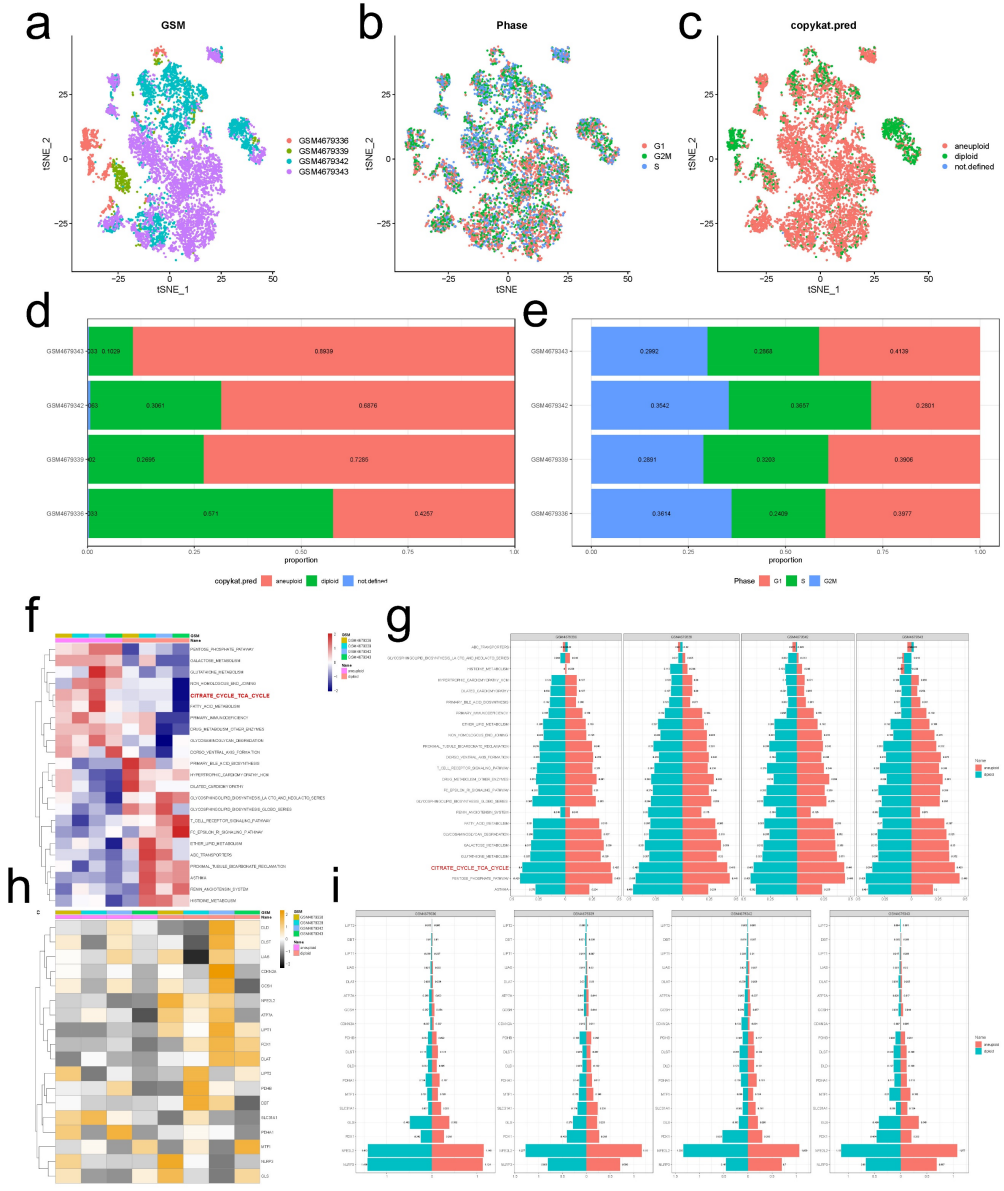
** Normal and malignant endometrial cells. (a)** A t-SNE map illustrating the distribution of cells in each EC sample, with each color representing a different sample's cells. **(b)** The t-SNE diagram displays the color-coded distribution of cell cycle characteristics. **(c)** The t-SNE diagrams of tumor and normal cells in EC samples are depicted with distinct hues. **(d)** The ratio of tumor cells to normal cells presents in each EC sample. **(e)** The proportion of cells in the G1, G2/M, and S phases in each EC sample. **(f, g)** The enrichment scores of different KEGG signal pathways in normal and malignant cells of each EC sample. **(g, h)** Difference in expression of CRGs between tumor and normal cells in EC.

Figure 1a depicts the TSNE diagram for the distribution of four GSM samples, while Figure 1b depicts the distribution of cells in various cell cycle phases. Figure 1c depicts that there were 5,911 tumor cells, 1,667 normal cells, and 32 unidentified cells. As demonstrated in Figure 1d, the proportion of tumor cells in the vast majority of EC samples was substantially greater than the proportion of normal cells. The preponderance of cells were in the G1 phase, and the proportion of cells in the G2M phase was nearly equal to that of cells in the S phase (Figure 1e). After segregating tumor cells from EC tissues, ssGSEA was used to calculate the enrichment scores of KEGG pathways at the level of a single cell. Our findings demonstrated that the citrate cycle TCA scores of tumor cells were considerably higher than those of normal cells (Figures 1f, g), suggesting that the EC may develop by mediating CRG-related processes. CRG-targeted interventions can induce tumor cell apoptosis and improve patient prognosis. Figures 1h, i depict the subpopulation distributions of CRG-related genes in solitary cells.

### Genetic and transcriptional change of CRGs

**Figure 2:**
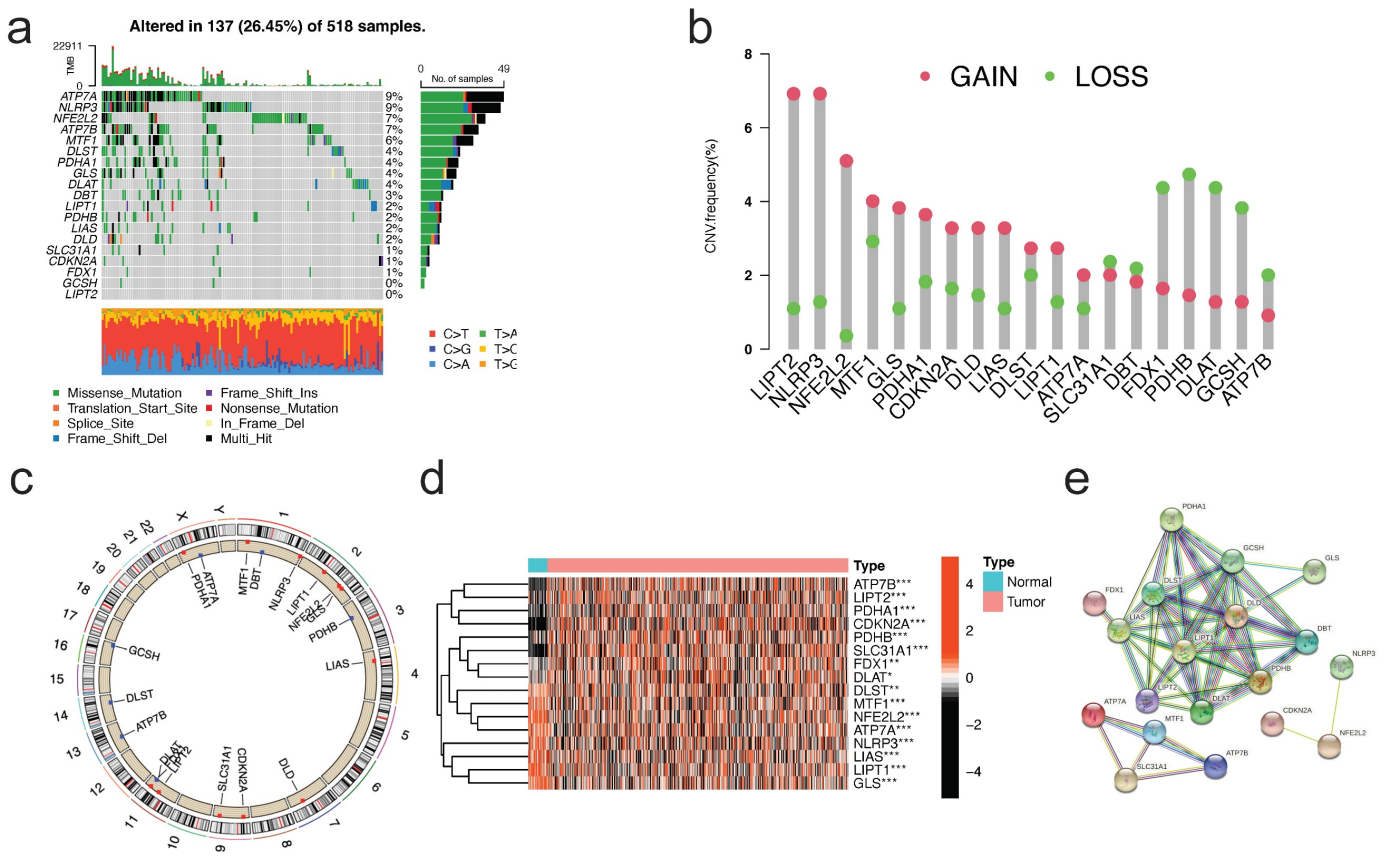
** Genetic and transcriptional alterations of CRGs in EC. (a)** Mutation frequencies of 19 CRGs in 518 EC patients from the TCGA cohort. **(b)**Frequencies of CNV gain, loss, and non-CNV among CRGs.** (c)** Locations of CNV alterations in CRGs on 23 chromosomes. **(d)** Expression distributions of 16 differentially expressed CRGs between normal and EC tissues. **(e)** PPI network showing the interactions of the CRGs (The minimum interaction score required for PPI analysis was set to 0.4 (moderate confidence)). CRGs, cuproptosis-related genes; EC, endometrial cancer; TCGA, The Cancer Genome Atlas; CNV, copy number variant.

Our summary analysis of the incidence of somatic mutations in 19 CRGs showed that 137 (26.45%) of 518 EC samples of TCGA had CRG mutations (Figure 2a). Among them, the mutation frequency of ATP7A was the highest (9%), followed by NLRP3, NFE2L2, ATP7B, MTF1, and DLSTT, while LIPT2 had no mutation. Next, we studied somatic copy number changes in these CRGs and found common copy number changes in all 19 CRGs. Among them, LIPT2, NLRP3, NFE2L2, MTF1, GLS, PDHA1 had extensive increase in copy number variation (CNV), while FDX1, PDHB, DLAT, GCSH, and ATP7B showed decrease in CNV (Figure 2b). The location of CNV changes in CRG on their respective chromosomes is shown in Figure 2c. We further compared the mRNA expression levels of 19 CRGs in EC and normal endometrial tissues, and found that 16 CRGs had significant differential expression, of which 8 genes were highly expressed in EC and 8 showed low expression (Figure 2d). In order to further explore the interaction of these CRGs, we conducted protein-protein interaction (PPI) analysis by String database (https://cn.string-db.org/). We found that there were extensive interactions among these CRGs (Figure 2e).

### Identification of cuproptosis subtypes

To fully understand the expression pattern of CRG involved in EC tumorigenesis, 492 patients with full clinical data from TCGA database were collected in our study for further analysis. The results of univariate Cox regression and Kaplan-Meier analysis revealed the prognostic values of 11 CRGs in patients with EC, determined the optimal cutoff value through the 'surv cutpoint' function, and p<0.05 was selected as the threshold for filtering (Figure S4). Next, we performed a multivariate Cox regression analysis on 11 prognostic CRGs, CDKN2A, PDHA1, GLS, DBT, and SLC31A1 were identified as independent predictive factors (Table 2).

**Figure 3:**
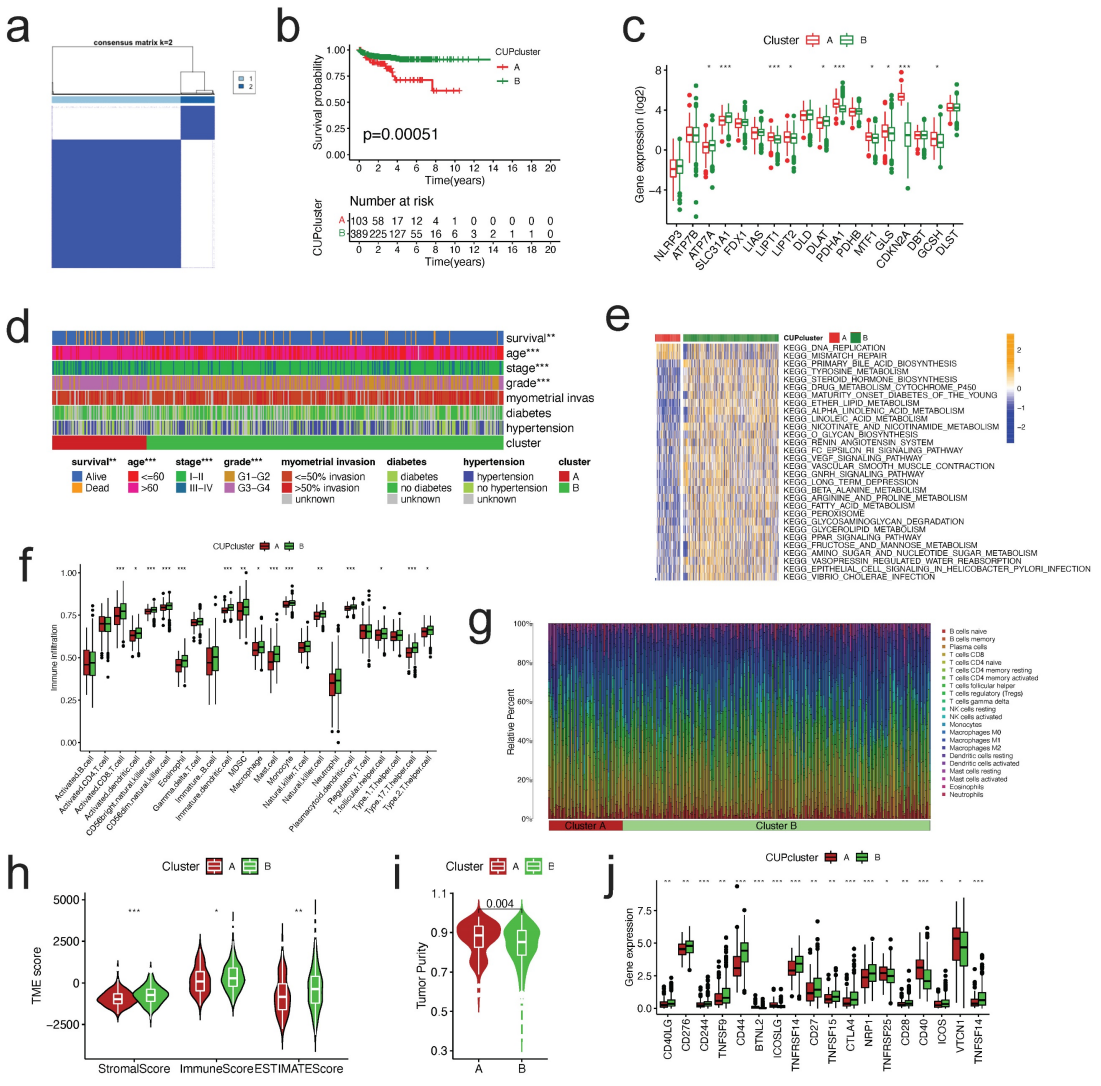
** Molecular subtypes constructed based on CRGs and their biological characteristics and TME differences. (a)**Consensus matrix of EC patients, k = 2, using the unsupervised consensus clustering approach. **(b)**Kaplan-Meier curve for overall survival of all EC patients with two cuproptosis subtypes (log-rank test, P =0.00051). **(c)**The 18 CRGs expression difference in EC patients stratified by two cuproptosis subtypes. **(d)**Differences between clinicopathologic features and two cuproptosis subtypes. **(e)**GSVA of KEGG biological pathways in two cuproptosis subtypes. Orange represents activation of biological pathways and blue represents inhibition of biological pathways, respectively. **(f)**Comparison of the ssGSEA scores for immune cells in the two cuproptosis subtypes. The line in the box represents the median value. **(g)**The relative percentage of subpopulations of immune cells in EC samples stratified by two cuproptosis subtypes. **(h)**Comparison between the TME score (stromal score, immune score, and Estimate score) and two cuproptosis subtypes. **(i)**Comparison between the tumor purity and two cuproptosis subtypes. **(j)**Expression levels difference of check-points genes in the two cuproptosis subtypes. TME, tumor microenvironment; EC, endometrial cancer; GSVA, gene set variation analysis; ssGSEA, single sample gene set enrichment analysis. The asterisk represents the p value (*p<.05, **p<.01, ***p<.001, similarly hereinafter).

To further explore the expression characteristics of CRGs in EC, we used a consensus clustering algorithm to categorize the patients with EC (Figure S5). According to the clustering criteria, we chose k = 2 to be an optimal selection for sorting the entire cohort (Figure 3a). Thus, two subtypes, designated Cluster A, and Cluster B, respectively, were identified, in which Cluster A included 103 cases, and Cluster B included 389 cases. Kaplan-Meier survival analysis revealed that overall survival (OS) differed obviously among the two subtypes, and Cluster A had noticeable survival worse preference (log-rank test, p = 0.00051, Figure 3b). Due to the rarity of GSE datasets including comprehensive clinical data for EC, despite the fact that the pathological type is sarcomas, we attempted to use GSE119041 to externally validate the repeatability of clustering. Again, we observed that two unique subgroups with relative dissimilar prognosis difference were clearly discovered (Figure S6). Most of the 18 CRGs in the two clusters displayed statistically significant expression differences (Figure 3c). In addition, the association between the two subtypes and a number of clinicopathological parameters (survival status, age, stage, grade, myometrial invasion, diabetes, hypertension) was explored, and survival status, age, and grade were found to have a clearly correlation with the clusters (Figure 3d).

### Characteristics of TME and biological function in different cuproptosis subtypes

GSVA enrichment analysis was done to explore the biological and functional differences between two subtypes (Figure 3e, Table S1). Cluster A was primarily enriched in carcinogenesis pathways, such as DNA replication and mismatch repair; Cluster B was primarily enriched in drug metabolism cytochrome p450, VEGF signaling route, PPAR signaling pathway, and other pathways. We examined the enrichment score of immune cells in the two categories using ssGSEA (Figure 3f). Cluster A suppressed the vast majority of immune cells, such as activated B cells, CD4/8 T cells, dendritic cells, CD56 dim natural killer cells, MDSC, mast cells, natural killer cells, Type 2/17 T helper cells, etc. To study further the variations in TME-infiltrating cell composition between the two clusters, the relative percentage of 22 types of immune cells was computed for each patient using the CIBERSORT method (Figure 3g). The findings of evaluating the TME score (stromal score, immune score, and estimate score) of the two clusters using the ESTIMATE package revealed that Cluster A patients had lower TME scores (Figure 3h). Regarding tumor purity, Cluster A has a higher score than Cluster B (Figure 3i). Clinically, targeted therapy research and development for malignant tumors is on the rise. We explored the association between the two clusters and the gene expression of common checkpoints and discovered that a number of checkpoint genes, including CD27/28/40/44/244/276, VTCN1, and TNFSF14, displayed substantial changes between the two clusters (Figure 3j).

### DEG-based identification of gene subtypes

**Figure 4:**
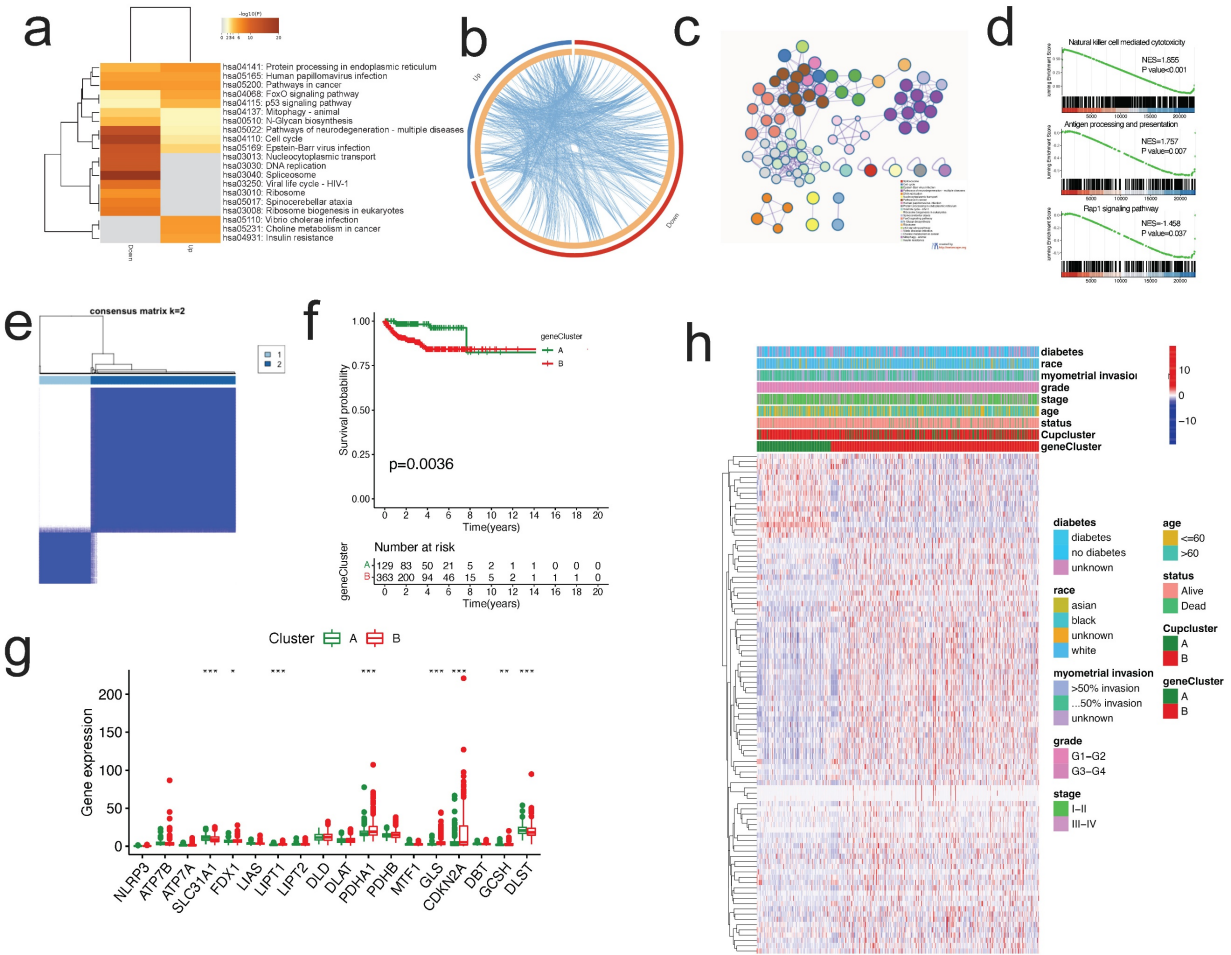
** Identification of gene subtypes based on the DEGs of cuproptosis-related clusters. (a-c)** KEGG enrichment analysis **(a)**, Circos plot **(b)**, and GO enrichment analysis **(c)** of DEGs by Metascape. **(d)** GSEA plots of DEGs. **(e)**Identification of gene subtypes based on prognostic DEGs among two cuproptosis subtypes. **(f)** Kaplan-Meier curves for overall survival with two gene subtypes (log-rank test, p =0.0036). **(g)** Differences in the expression of 18 CRGs among the two gene subtypes. **(h)** Heatmap showing the relationships between clinicopathologic features, cuproptosis subtypes, and the two gene subtypes. DEGs, differentially expressed protein-coding genes; GO, Gene Ontology; CRGs, cuproptosis-related genes.

To examine the probable biological function of each cuproptosis subtype in EC, the "limma" R package was used to detect the DEGs between the two clusters, resulting in the identification of 2,152 DEGs associated to cuproptosis subtypes (Table S2). The DEGs were significantly abundant in cell cycle and carcinogenesis-related pathways, such as cell cycle, platinum drug resistance, cancer pathways, p53 signaling, DNA replication, and AMPK signaling pathway, according to KEGG enrichment analysis (Figure 4a). Circos plot found extensive relationships between gene groups with high and low expression differentials (Figure 4b). Additionally, GO enrichment analysis found that DEGs are enriched in the cell cycle, DNA replication, p53 pathway, and other categories (Figure 4c). According to GSEA, immune-related pathways were enriched, including natural killer cell mediated cytotoxicity, antigen processing and presentation, and Rap1 signaling pathway (Figure 4d).

Then, we determined the predictive significance of DEGs using univariate Cox regression analysis and identified 75 genes related with overall survival (p<0.001) for further study. To further examine the regulation mechanism of the 75 prognostic genes, EC patients were divided using a consensus clustering method in order to investigate the process (Figure S7). We selected k = 2 to split all EC patients into gene subtypes A and B. (Figure 4e). Subsequent analysis of OS indicated a statistically significant difference between the two gene subtypes (log-rank test, p = 0.0036, Figure 4f). As predicted, the expression of the CRGs differed substantially between the two gene clusters (Figure 4g). Additionally, the link between the two gene clusters and other clinicopathological variables, including clusters of cuproptosis, was examined (Figure 4h).

### Construction the CRGPI in the training cohort

**Figure 5:**
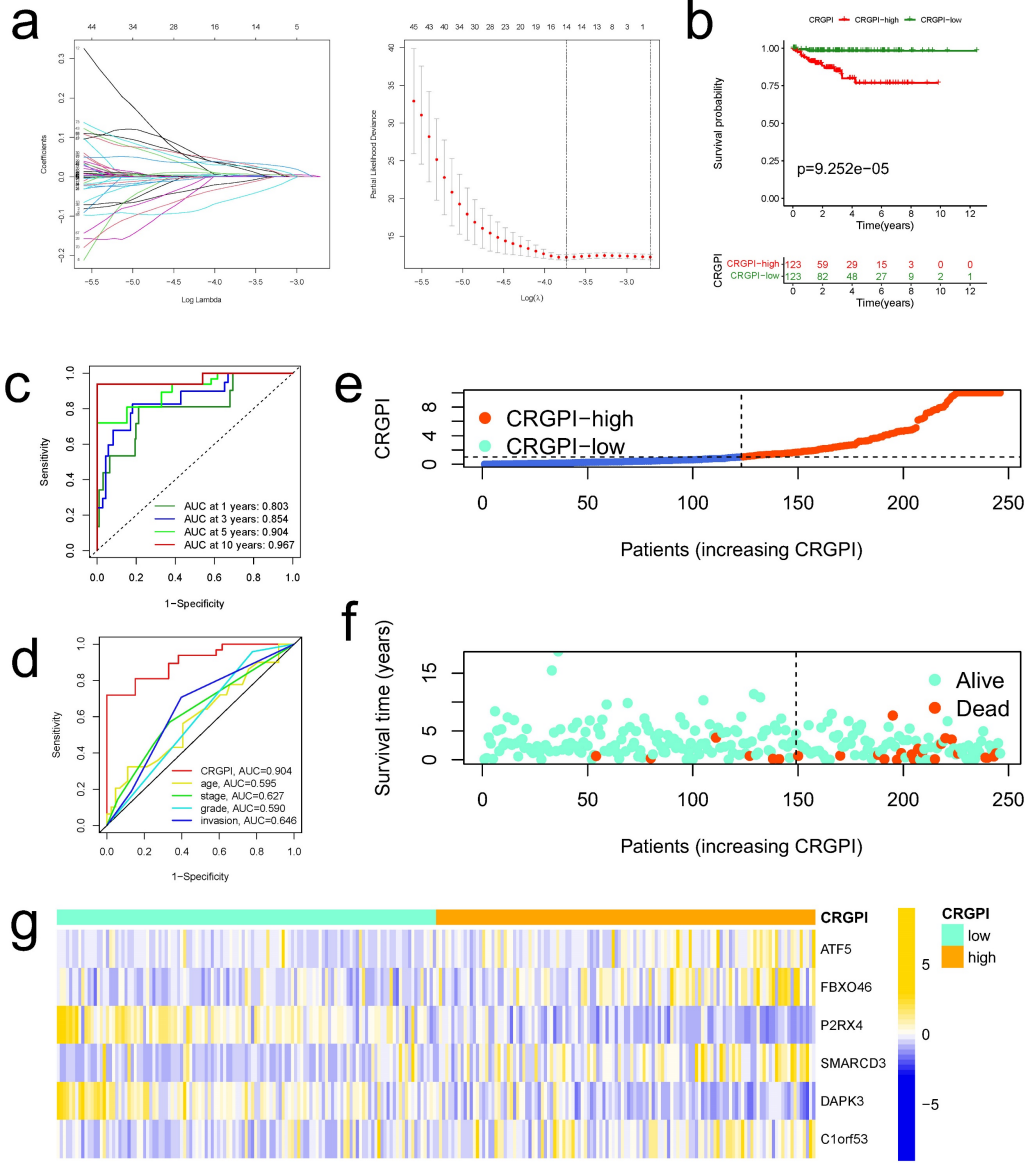
** Construction of CRGPI in training cohort. (a)**The LASSO regression analysis and partial likelihood deviance on the prognostic genes. **(b)**Kaplan-Meier analysis of the overall survival between the CRGPI-high and CRGPI-low groups in the training cohort. **(c)**ROC curves and their AUC values for CRGPI represented 1-, 3-, 5-, and 10-year predictions. **(d)**ROC curves of CRGPI and clinicopathological factors (age, grade, stage, and myometrial invasion) for 5-year AUC. **(e)**Scatter plot showing the correlation between the survival status and CRGPI. **(f)**Risk score distribution plot showing the distribution of the CRGPI-high and CRGPI-low. **(g)**Heatmap showing expression of the six genes between the CRGPI-high and CRGPI-low groups.

The CRGPI was established based on the prognostically relevant DEGs in the training group. First, Lasso analysis suggested 14 genes based on the minimum partial likelihood deviation (Figure 5a); Next, we performed multivariate Cox regression analysis on these genes, which revealed that six genes, including ATF5, FBXO46, P2RX4, SMARCD3, DAPK3, and C1orf53, were independent prognostic factors for patients with EC. Table S2 contains the correlation coefficients. Our CRGPI is computed based on the results of a multivariate Cox regression analysis as follows:

CRGPI = 0.0075*ATF5 + 0.1072*FBXO46 + -0.2219*P2RX4 + 0.0767*SMARCD3 + -0.1188*DAPK3 + 0.06680*C1orf53.

Based on the median CRGPI, patients were separated into two groups: those with a high CRGPI and those with a low CRGPI. The CRGPI-high group in the training cohort had a poorer prognosis, as evidenced by Kaplan-Meier curves (Figure 5b). Using the ROC curve, the accuracy of the prognostic signature was determined; the AUC values for 1, 3, 5, and 10 years of OS were 0.803, 0.854, 0.904, and 0.967, respectively (Figure 5c). The predictive signature exhibits the highest 5-year AUC values relative to other clinicopathological variables (age, stage, grade, and myometrial invasion) (Figure 5d). As illustrated by the scatter plot, EC patients with a high CRGPI had a shorter survival rate than those with a low CRGPI (Figure 5e). The CRGPI distribution map corresponded to patient group categorization (Figure 5f). In addition, the heatmap revealed substantial differences between CRGPI-high and CRGPI-low groups in the expression of six CRGPI genes (Figure 5g).

### Internal and External Validation of the CRGPI

To validate the predictive value of the CRGPI, CRGPIs of patients in testing cohorts and overall cohorts were calculated, and patients were categorized into CRGPI-low and CRGPI-high groups based on the median CRGPIs in the training cohorts. The Kaplan-Meier and ROC analysis of OS survival in the testing and total cohorts revealed that these results were comparable to those of the training cohorts (Figure S8a-c, g-i). These results verified the CRGPI further. According to the CRGPI distribution map, the survival rate of the CRGPI-high group was less than that of the CRGPI-low group (testing cohorts, Figures S6d, e; overall cohorts, Figure S8j, k). The heatmaps illustrate the expression profiles of six genes in the training cohorts (testing cohorts, Figures S6f; overall cohorts, Figure S8l).

**Figure 6:**
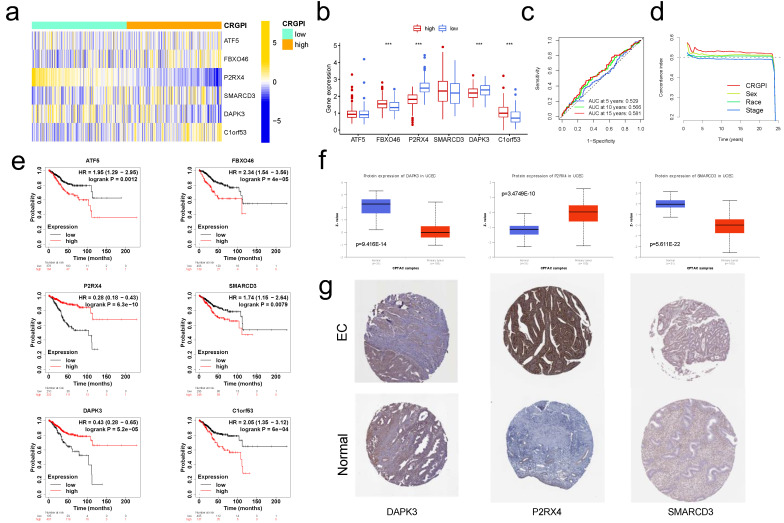
** External validation for CRGPI. (a)**Heatmap showing expression of the six genes between the CRGPI-high and CRGPI-low groups in IMvigor210 datasets. **(b)**Boxplot showing the expression difference of six genes between the CRGPI-high and CRGPI-low groups in IMvigor210 datasets. **(c)**ROC curves and their AUC values for CRGPI represented 5-, 10- and 15-year predictions in in IMvigor210 datasets. **(d)**Concordance index curves of CRGPI and clinicopathological factors (sex, race, and stage) for 0-25 years in IMvigor210 datasets. **(e)**Kaplan-Meier survival analysis plot of each CRGPI gene in CRGPI by KM plotter database. **(f)**The boxplot showing the difference in protein expression of 3 CRGPI genes: DAPK3, P2RX4, and SMARCD3, according to CPTAC database; **(g)**The typical protein expression figures by immunohistochemistrical staining for 3 CRGPI genes: DAPK3, P2RX4, and SMARCD3, according to HPA database.

Using the "IMvigor210" dataset containing clinical information and RNA-seq data from metastatic urothelial cancer patients treated with the ICI atezolizumab (PD-L1 inhibitor) 19, we further validated this observation. Using the correlation coefficient to construct the CRGPI, the IMvigor210 cohort was divided into high and low CRGPI groups. Patients with a low-CRGPI had a clearly better prognosis (Figure 10e), and there was preliminary evidence that patients with a low-CRGPI had a better immunotherapy outcome than those with a high-CRGPI. Figure 6a depicts the consistent expression profiles of six CRGPI genes in the IMvigor210 cohorts using a heatmap. The majority of gene expression was significantly different between the high and low groups, and the trend reported in EC was replicated (Figure 6b). Figure 6c displayed the AUC values for 5-, 10-, and 15-years of OS, whereas Figure 6d displayed the concordance index curves of CRGPI. These outcomes revealed that our CRGPI had a reliable prognostic and predictive potential.

Using the KM plotter database, we investigated the predictive effect of the six CRGPI genes in 542 EC patients. High expression levels of ATF5, FBXO46, SMARCD3, and C1orf53 were seen to be associated with a poorer OS, whereas low expression levels of P2RX4 and DAPK3 were observed to be related with a poorer OS (Figure 6e). These findings aligned with the trend reported in the TCGA database.

Using the CPTAC database (http://ualcan.path.uab.edu), we examined the expression of prognostic proteins in EC and normal endometrial tissue. Figure 6f demonstrates that the expression levels of DAPK3, P2RX4, and SMARCD3 in EC were significantly different from those in normal endometrial tissue (all p<0.001). In the HPA database (https://www.proteinatlas.org), the protein expression of DAPK and SMARCD3 in endometrial tissue was medium to low, while their expression in EC was low; the protein expression of P2RX4 in EC was high to medium, while its expression in endometrial tissue was low to undetectable (Figure 6g).

In order to further illustrate the predictive ability of our CRGPI, we examined and compared four published EC prognostic models with our own. Compared to Shan's cuproptosis-related subgroup's gene signature 28, Liu's ferroptosis-related gene signature 29, Liu's EMT-related gene signature 30, and Wang's immune-related gene signature 31, survival curves revealed that the prognosis for high-risk group patients was significantly worse in all of these models (all p < 0.05, Figure S9a). According to the ROC curve, the AUC values for these four models were less than ours (Figure S9b). The time-dependent AUC plot and concordance index plot provide additional support (Figure S9c, d). These results suggested that the efficacy of our CRGPI appeared superior to that of other studies previously reported.

### Analysis of clinical correlation and stratification of the CRGPI

**Figure 7:**
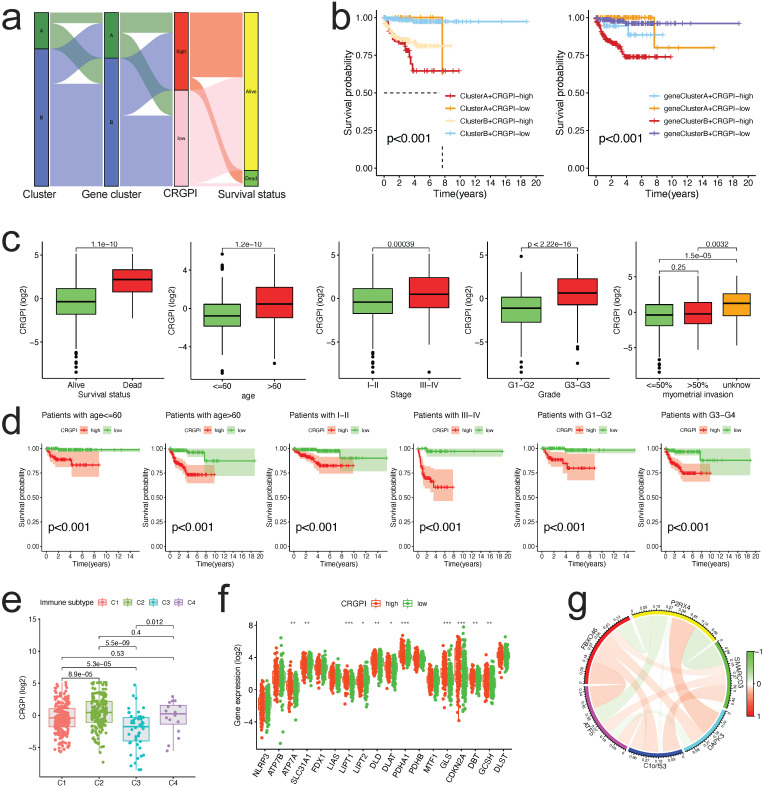
** Correlation between CRGPI and clinicopathological variables. (a)**An alluvial representation of the distribution of the cuproptosis cluster, gene cluster in two CRGPI groups, and survival outcomes. **(b)**The K-M survival curves were stratified according to cuproptosis cluster and CRGPI subgroup (left panel), as well as gene cluster and CRGPI subgroup (right panel). **(c)**A comparison of clinicopathological variables (survival, age, stage, grade, and myometrial invasion) with CRGPI. **(d)**The K-M survival curves for two CRGPI groups were stratified by age (<=60 and >60 years), stage (Stage I-II and Stage III-IV), and grade (G1-G2, G3-G4). **(e)**Difference of CRGPI among the immune subgroups (C1-Wound Healing, C2-IFN-gamma Dominant, C3- Inflammatory, C4-Lymphocyte Depleted). **(f)**The CRGs expression difference in EC patients stratified by CRGPI subtypes. **(g)**The co-relationship of six proteins in the CRGPI.

The alluvial diagram was applied to represent the survival differential between the different cuproptosis clusters, gene clusters, CRGPI, and survival status with greater clarity (Figure 7a). The majority of cuproptosis cluster A patients also belonged to the CRGPI-high group, which had the poorest survival outcome; similarly, gene Cluster B with CRGPI-high patients likewise had the poorest survival outcome (Figure 7b). The correlation between the CRGPI and clinicopathological variables was then examined. Myometrial invasion did not correlate with CRGPIs (Figure 7c). All stratified clinicopathological parameters (age, stage, and grade, all p < 0.001, Figure 7d) could be accurately predicted using the CRGPIs.

Furthermore, based on the results of immunotyping of pancancer in the literature 32, we compared the relationship between CRGPI and immunotyping of EC and discovered that there were significant differences between the four existing immune subtypes C1 (wood healing), C2 (IFN gamma dominant), C3 (inflammatory), and C4 (lymphocyte completed) and CRGPI in the TCGA dataset of EC, indicating a potential relationship between our CRGPI and immune subtypes (Figure 7e). As expected, there were distinct changes in CRG expression across groups with high and low CRGPI (Figure 7f). The majority of prognostic signature proteins are positively associated, as shown by their correlation analysis (Figure 7g).

### Association between the CRGPI and tumor infiltrating immune cells

**Figure 8:**
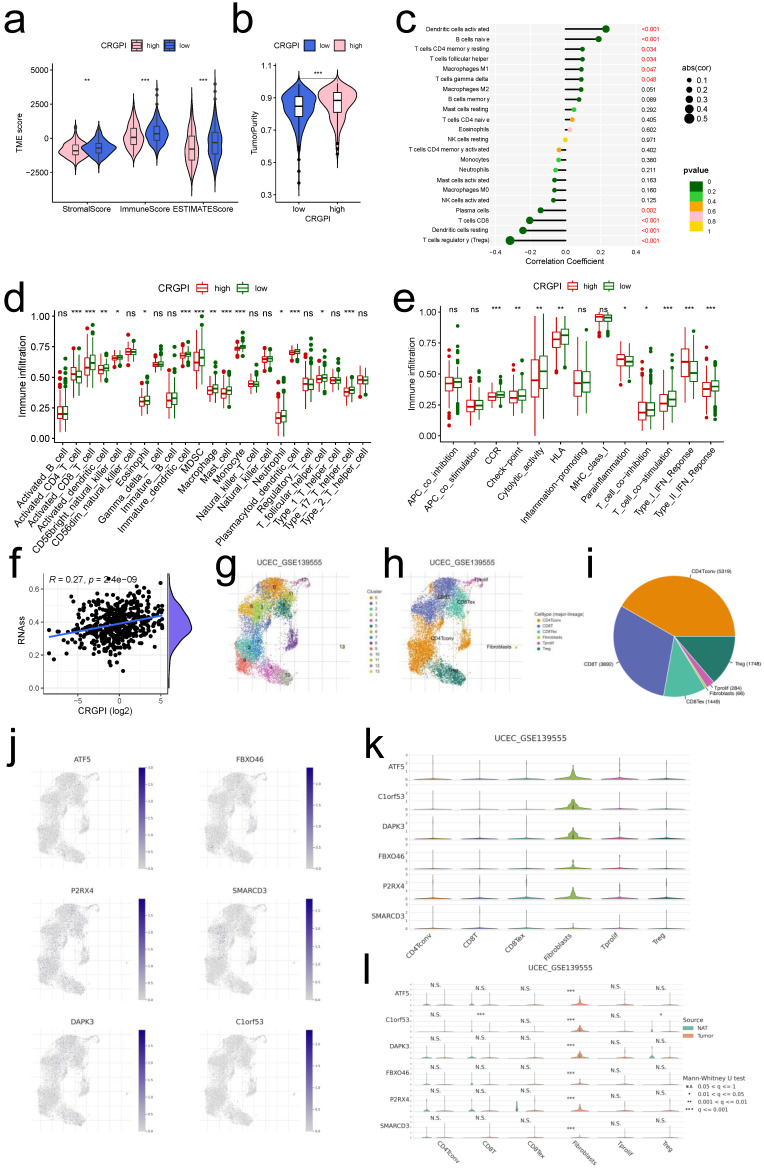
** The landscape of immune microenvironment with CRGPI. (a)**Correlations between CRGPI and immune score, stromal score, and ESTIMATE score. **(b)**Correlations between CRGPI and tumor purity. **(c)**Correlations between CRGPI and immune cell type based on CIBERSORT. **(d-e)**. Comparison of the ssGSEA scores for immune cells **(d)** and immune functions **(e)** for patients between the CRGPI-high and -low groups. The line in the box represents the median value. **(f)** The linear correlation between CRGPI and cancer steam cell index. **(g-i)** The cell types and their subgroup distribution in UCEC GSE13955 dataset; **(j)** Distribution of CRGPI six genes in different cells in UCEC GSE13955 dataset; **(k)** Distribution of six CRGPI gene's expression in different cell types using violin plot in UCEC GSE13955 dataset; **(l)** Distribution of six CRGPI gene's expression in different cell types of normal endometrial and tumor cells in UCEC GSE13955 dataset.

Previous findings indicated that our CRGPI may be associated with tumor immunity. Comparing the TME scores of each CRGPI group, we found that the CRGPI-high group had considerably lower TME scores (all p < 0.01, Figure 8a) and a significantly higher score for tumor purity (p < 0.001, Figure 8b). CRGPI was positively correlated with active dendritic cells, B cells naive, resting T cells CD4 memory, and T cells follicular helper, but negatively correlated with regulatory T cells, resting Dendritic cells, T cells CD8, and plasma cells (all p < 0.05, Figure 8c). Many immune cells (Figure 8d) and immunological functions (Figure 8e) exhibited notable differences between the two CRGPI groups, with the CRGPI-low group displaying a greater number of more immune cells and immunological functions. In addition, CRGPI was strongly positively linked with cancer stem cells (R = 0.27, p < 0.001, Figure 8f), indicating that samples with higher scores displayed more pronounced stem cell features and less cell differentiation. Overall, these data revealed that the CRGPI reflected the degree of immune cell and tumor microenvironment (TME) infiltration; the level of immune infiltration was lower in the CRGPI-high group, which had a poorer prognosis; immune infiltration is responsible for adaptive antitumor immunity.

### Single-Cell level analysis of CRGPI

Herein, we intended to localize six CRGPI genes at the level of a single cell in order to examine their possible link with immune cells. By examining the TISCH2 database, we observed that all six genes were expressed in immunological single cell subpopulations (Figure 8g-l).

Six cell types were identified in UCEC GSE13955, including CD4Tconv, CD8T, CD8Tex, Fibroblasts, Tprolif, and Treg, with CD4Tconv cells exhibiting the highest cell numbers (Figure 8g-i). And all of the six cell types exhibited six CRGPI gene expression (Figure 8j), with the Fibroblasts subgroup cells displaying the greatest abundance of all six genes (Figure 8k), as well as the greatest number of tumor cells relative to normal endometrial cells (p < 0.001, Figure 8l). Four cell types were identified in UCEC GSE154763, including DC, Mast, Mono/Macro, and pDC, while Mono/Macro cells exhibited the most abundant cell counts (Figure S10a-c). And all four cell types expressed six CRGPI genes (Figure S10d-f), with ATF5 being predominantly expressed in pDC cells, DAPK3 and SMARCD3 being highly elevated in Mast cells, and P2RX4/C1orf63 being largely expressed in Mono/Mast cells (Figure S10e). ATF5 was significantly expressed on Mono/Macro and pDC subgroups in tumor cells, whereas C1orf53 was predominantly expressed on Mono/Macro subgroup and SMARCD3 was predominantly expressed on Mast subgroup (Figure S10f). The foregoing single-cell investigations demonstrated that the CRGPI genes were highly expressed in all immune cell subsets of endometrial cancer, thus validating the relationship between CRGPI and TME.

### CRGPI correlation with TMB and MSI

**Figure 9:**
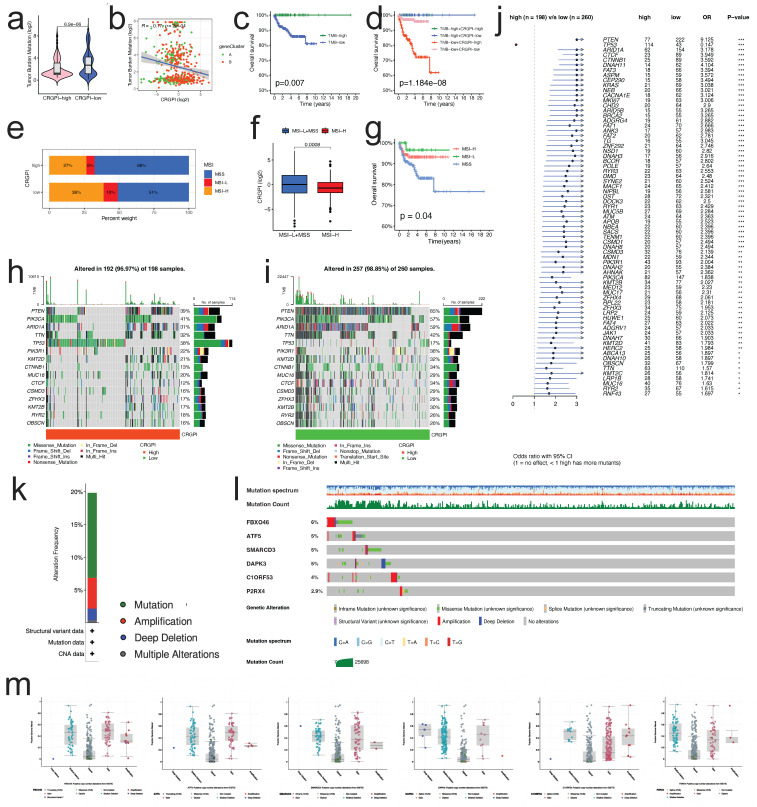
** Mutation spectrum and MSI analysis of the patients between two groups. (a)** The TMB of CRGPI-high group was significantly lower than that of CRGPI-low group. **(b)** The scatterplot depicted the negative correlation between CRGPI and TMB. **(c)** KM curves of overall survival in different TMB subgroups. **(d)** KM curves of overall survival stratified by both TMB and CRGPI. **(e)** The differences in the percent of MSI-H, MSI-L, and MSS status between CRGPI-high and -low groups. **(f)** The CRGPI of MSI-H group was significantly lower than that of MSS/MSI-L group. **(g)** KM curves of overall survival in different MSI subgroups. **(h, i)** The waterfall diagram showed that top 15 driver genes exhibiting the highest mutation frequency in CRGPI-high group (h) and CRGPI-low group **(i)**. **(j)** Forest plot showed the genes that exhibit significant differences in mutational rate between the two groups. **(k)** Frequency of mutation in six CRGPI genes in uterus endometrial cancer. **(l, m)** Mutation of each gene.

The TMB of the CRGPI-high group was significantly lower than that of the CRGP-low group, as indicated by the results (p < 0.001, Figure 9a). The correlation analysis of CRGPI and TMB revealed a significant negative correlation (R = -0.25, p < 0.001, Figure 9b) between the two variables. Next, we analyzed the TMB's effect on prognosis and found that elevated TMB was associated with better prognoses in EC patients (Figure 9c). Considering the synergistic effect of TMB and CRGPI, their effect on prognostic stratification was assessed. As depicted in Figure 9d, the survival difference of CRGPI subtypes was statistically significant in both high and low TMB groups (p < 0.001), with the poorest prognosis belonging to low TMB patients in the CRGPI high group. By analyzing the relationship between the CRGPI and MSI status, we determined that 39% of MSI-H patients were in the CRGPI-high group and 27% were in the CRGPI-low group (Figure 9e). Furthermore, the CRGPI was significantly lower in MSI-H patients than in MSS and MSI-L patients (Figure 9f, p < 0.001). MSI-H patients had a relatively better prognosis compared to MSS patients (P = 0.04, Figure 9g). In addition, we evaluated differences between CRGPI-high and CRGPI-low groups regarding somatic variation driving genes. We mapped the top 15 driving genes with the highest mutation frequency in the CRGPI high and low groups using the waterfall diagram (Figure 9h, i). The mutation frequency of many genes, including TP53, PTEN, ARID1A, CTCF, CTNNB1, and DNAH11 differed significantly between the CRGPI-high and -low groups, as revealed by an analysis of the mutation annotation files (Figure 9j). What's more, we searched the mutation status of six CRGPI genes on the cBioPortal website (Figure 9k). The gene mutation frequencies from high to low are FBXO46, ATF5, SMARCD3, DAPK3, C1orf53, and P2RX4 (Figure 9l). Among them, missense mutation was the most common type of mutation, followed by amplification, deep deletion and finally multiple alterations (Figures 9l, m).

TMB and MSI status are well-known to be promising predictive biomarkers for immune checkpoint inhibitor treatment 8, 33. These results suggest that the CRGPI was clearly associated with TMB and MSI status, which may provide novel ideas for investigating targeted therapy and immunotherapy based on the CRGPI composition and gene mutation.

### Predicting immunotherapeutic benefits

**Figure 10:**
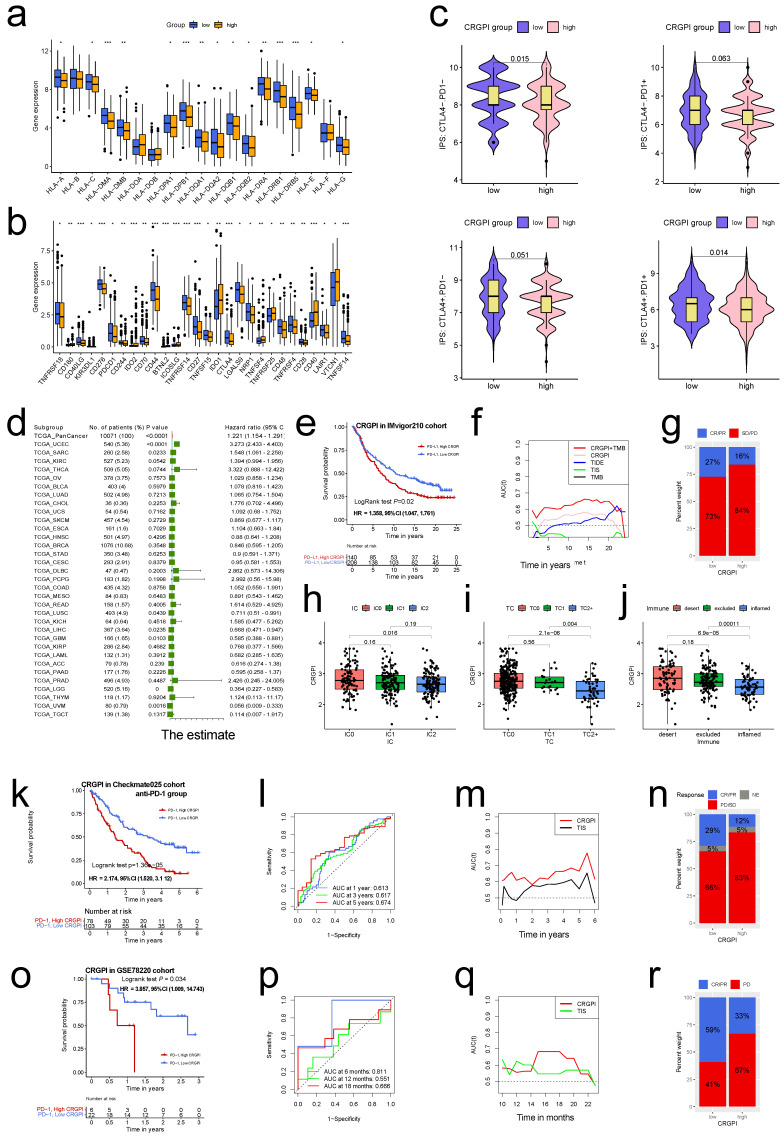
** The CRGPI is a prognostic biomarker and predict patients' immunotherapeutic benefit. (a)**The expression of HLA family members between high and low CRGPI in EC patients of TCGA cohort; **(b)**The expression of immune checkpoints between high and low CRGPI in EC patients of TCGA cohort; **(c)**The association between IPS and the CRGPI in EC patients of TCGA cohort; **(d)**Subgroup analyses estimating prognostic value of CRGPI in different cancer types from TCGA data sets. The length of horizontal line represents the 95% confidence interval for each group. The vertical box line represents the HR of all patients. **(e)**K-M survival analysis of the CRGPI subgroups in IMvigor210 cohort; **(f)**The time-dependent AUC showing the comparison of CRGPI, TIDE, TIS, and TMB in time range from 0 to 25 years in IMvigor210 cohort; **(g)**Proportions of anti-PD-L1 immunotherapy response in high and low CRGPI groups in IMvigor210 cohort; **(h-j)** The difference of CRGPI among PD-L1 expression of different IC (h), TC (i), and immune subtypes (j) in the IMvigor210 cohort. **(k)** K-M survival analysis of the CRGPI subgroups in CheckMate025 cohort; **(l)** ROC curves and their AUC values for CRGPI in 1-, 3-, and 5-year predictions in in CheckMate025 cohort; **(m)** The time-dependent AUC showing the comparison of CRGPI, and TIS in time range from 0 to 6 years in CheckMate025 cohort; **(n)** Proportions of anti-PD-1 immunotherapy response in high and low CRGPI groups in CheckMate025 cohort; **(o)** K-M survival analysis of the CRGPI subgroups in GSE78220 cohort; **(p)** ROC curves and their AUC values for CRGPI in 6-, 12-, and 18-month predictions in in GSE78220 cohort; **(q)** The time-dependent AUC showing the comparison of CRGPI, and TIS in time range from 0 to 24 months in GSE78220 cohort; **(r)** Proportions of anti-PD-1 immunotherapy response in high and low CRGPI groups in GSE78220 cohort; PR, Partial Response; PD, Progressive Disease; SD, Stable Disease; CR, Complete Response; IC, tumor-infiltrating immune cells; TC, tumor cells.

In light of the significance of immunotherapies based on HLA and checkpoint inhibitors, we evaluated the differences in HLA family members and immune checkpoint expression between the two CRGPI groups. Significant differences were observed in the expression of HLA members, with the majority of HLA members exhibiting high expression in CRGPI low group (Figure 10a); with regard to checkpoints, we observed that numerous genes exhibited differential expression between two groups, with CTLA4, PDCD1 exhibiting high expression in CRGPI low group (Figure 10b).

Most HLA and checkpoint genes were broadly connected with the CRGPI six genes, with FBXO46 being primarily negatively correlated (Figure S11a), and ATF5 being primarily positively correlated (Figure S11b).

Using Immunophenoscore, we subsequently evaluated the response to immune checkpoint inhibitors (CTLA4-PD1-, CTLA4-PD1+, CTLA4+PD-, and CTLA4+PD-) in subgroups stratified by CRGPI. Figure 10c demonstrates that the CRGPI-low group had a bigger IPS, indicating a higher immunogenicity of tumors and a greater responsiveness to ICI. We examined the predictive significance of the CRGPI in 33 distinct TCGA cancer cohorts with 10071 tumors (Table S3). The CRGPI was supported as a favorable prognostic biomarker in seven independent TCGA cohorts (Figure 10d), including endometrial cancer, brain lower grade glioma, uveal melanoma, glioblastoma multiforme, sarcoma, liver hepatocellular carcinoma, and lung squamous cell carcinoma, despite heterogeneous subgroup analysis results.

Monoclonal anti-bodies that block the T-cell inhibitory molecules PD-L1 and PD-1 have emerged as an anticancer treatment with exceptional and synergistic survival effects.

Next, we investigated the predictive utility of the CRGPI for immune-checkpoint therapy by separating patients in the IMvigor210, Checkmate025 (anti-PD-1 group), and GSE78220 cohorts into high and low CRGPI groups. In the IMvigor210 cohort (HR, 1.358; 95% CI, 1.047, 1.761; Figure 10e), Checkmate025 cohort (HR, 2.174; 95%CI, 1.520, 3.112; Figure 10k), and the GSE78220 cohort (HR, 3.857; 95% CI, 1.009, 14.74; Figure 10o), patients with a high CRGPI had significantly shorter overall survival than those with a low CRGPI. The predictive value of the CRGPI to checkpoint immunotherapy was also confirmed in IMvigor210 (Figure 10f-j), Checkmate025 (Figure 10l-n) and GSE78220 (Figure 10p-r). The relationship between the CRGPI and the IC, TC, and immune types was analyzed, revealing that the CRGPI of IC2 was lower than that of IC0 (Figure 10h), that TC2 had a lower CRGPI than the other two groups (Figure 10i), and that the immune-inflamed type has the lowest CRGPI compared to the immune-desert and immune-excluded types (Figure 10j). The CRGPI was negatively correlated with the PD-L1 expression level, and a low CRGPI was strongly associated with the immune-inflamed subtype.

The predictive utility for CheckMate025 was further validated in its merged group (Figure S12a), but it's not significant for anti-mTOR inhibitor subgroup (Figure S12b), this is concordance with the conclusion that OS benefit with nivolumab over the everolimus 25. The similar trend was also observed as to the progression-free survival (Figure S12 c-e).

We assessed the clinical utility of CRGPI in response to immunotherapy. Patients with lower CRGPI were more likely to benefit from immune-checkpoint therapy (IMvigor210 cohort: two-sided, P=0.024; Figure 10g; CheckMate025 cohort: P=0.003, Figure 10n). Although the difference was not statistically significant, the overall trend was similar (GSE78220, two-sided Fisher exact test, P=0.372, Figure 10r).

TMB, a promising predictive biomarker for efficacy of immunotherapy, was also assessed in the IMvigor210 cohort. We did not find any predictive advantage of TMB over CRGPI, but combining TMB and CRGPI considerably enhanced the predictive value above TMB or CRGPI alone (Figure 10f). Similar to TCGA (Figure 9d), the survival advantage of patients in the high CRGPI group was lower than that of the low CRGPI group for both high and low TMB groups (Log-rank test, P <0.001; Figure S12 f). ROC analyses of the CheckMate025, and GSE78220 cohorts also revealed that the CRGPI was a biomarker predictive of immunotherapeutic benefits (Figure 10l, p). Moreover, time-dependent ROC demonstrated that our CRGPI has a continuous prognostic advantage over TIDE, TIS scores, and TMB in TCGA dataset (Figure S12g) and other immunotherapy cohorts (Figure 10f, m, q).

Taken together, our data demonstrate that CRGPI is linked with response to immunotherapy strategies (anti-PD-1/ PD-L1), patients in the CRGPI-low group may have a stronger immunotherapy response.

### Predicting chemotherapy and molecular drug sensitivity in patients with EC

We examined the relationship between the CRGPI and sensitivity to chemotherapy and targeted therapy drugs in EC patients using the "pRRophetic" R package. Our results revealed that the estimated IC50 values of 59 different drugs differed significantly between the two CRGPI subgroups (all p < 0.01, Table S4). 17 drugs were more sensitive in the low CRGPI group than in the high group, including Mitomycin C, PARP inhibitor (ag-014699), 5-Fluorouracil, CDK inhibitor (Roscovitine), Bleomycin (Figure S13a); 42 drugs were more sensitive in the high CRGPI group than in the low group, including p38 MAPK inhibitor (KIN001-266), tyrosine kinase inhibitor (Masitinib), EGFR antibody (Cetuximab, Gefitinib), JNK inhibitor (JNK-9L) (Figure S13b).These findings suggested that the CRGPI could be used to predict chemotherapy and targeted therapy.

## Discussion

Endometrial cancer (EC) is the most common gynecologic malignancy in Western and Asian countries and a significant immunogenic cancer for which immune checkpoint inhibitors (ICIs) are clinically effective 34. According to The Cancer Genome Atlas Network, a comprehensive genomic analysis of primary EC identified four distinct molecular subgroups: "POLE ultramutated" (POLE), "hypermutated/microsatellite-unstable" (MSI-H), "copy number low/microsatellite-stable" (CN-L), and "copy number high" (CN-H) 35. Among these subtypes, EC with the first two subtypes is highly immunogenic and caries a greater number of novel antigens, causing an increase in tumor-infiltrating lymphocytes and a compensatory upregulation of immune checkpoint molecules 34, 36. Immunotherapy in EC has consequently received considerable attention. Immunotherapy, specifically ICIs, is the cutting-edge treatment for a number of solid malignancies, including gynecological cancers 37. In 2019, pembrolizumab and levatinib will be used to treat MS stable (MSS) diseases 38. In 2017, pembrolizumab was used to treat MSI-H tumors. In 2021, the European Medical Agency (EMA) also approved pembrolizumab, lenvatinib, and dostarlimab for pre-treatment EC patients and MSI-H EC patients, respectively. In light of the fact that the overall response rate to ICI treatment is still low 39, it is crucial to determine which patients stand to benefit the most from these treatments. Tsvetkov et al. 3 shed light on a new form of cell death, cuproptosis, which could provide a new avenue for anticancer treatments by fully exploiting copper's pathophysiological role. In this study, we investigated the variation and expression of CRGs in EC, as well as their impact on patient prognosis. Analysis of single-cell RNA data revealed that the TCA pathways were significantly upregulated in malignant cells compared to normal cells, indicating that copper-induced cell death may play a role in tumorigenesis in EC and that tumor cells may progress via upregulation of cuproptosis activities. CDKN2A, PDHA1, GLS, DBT, and SLC31A1 were independent prognostic risk factors for EC patients. The preponderance of CRGs were maladjusted in EC patients. We identified two molecular subgroups of EC based on CRG datasets, and this was confirmed by a GEO dataset of uterine carcinosarcomas. Subtype A patients had more advanced clinicopathological characteristics and a worse overall survival rate than subtype B patients. In addition, the characteristics of the TME and immune activation varied considerably between the two subtypes. Based on the DEGs between the two subtypes, we identified two subtypes of genes. In addition, we demonstrated the predictive ability of the robust and effective prognostic CRGPI based on six DEGs.

Comparison with the immunotyping constructed by Thorsson et al. 32 provided additional confirmation that our CRGPI was related to immunity; therefore, to gain further insight into the immunological nature of the CRGPI subgroups, we screened the various CRGPI subgroups for mutations in genes where missense mutations were the most prevalent type. PTEN was more prevalent in the CRGPI-low group (85% vs. 39%), whereas TP53 was more prevalent in the CRGPI-high group (58% vs. 17%). PTEN interacts with p53 to promote tumor progression 40, and not only is TP53 mutation the most common genetic event in cancer, but it is also associated with more aggressive and inferior clinical outcomes in a variety of cancers, including EC 41. In addition, there were fewer PIK3CA mutations in the CRGPI-high group (41% vs. 57%), suggesting that CRGPI-high ECs promote carcinogenesis via the PI3K-PTEN-AKT signaling pathway 41.

Next, we examine the relationship between CRGPI and HLA family genes and known predictive biomarkers for immunotherapy, such as immune checkpoint gene expression (PD1, PD-L1), TMB, MSI-H, and tumor-infiltrating lymphocyte (TIL) numbers 42, 43. The ability of T lymphocytes to recognize neoantigens via HLA molecules on the surface of tumor cells permits the initiation of targeted and efficient anti-cancer immune responses 44. Our results demonstrated that HLA genes' expression were significantly higher in the group with a low CRGPI. PD-1/PD-L1 status may impact the efficacy of ICI, and PD-L1-positive tumors tend to respond better to anti-PD-1/PD-L1 therapies than PD-L1-negative tumors in general 45; however, this notion has not been confirmed in ECs, and relevant experimental studies are awaiting. In our CRGPI-low group, the expression of a number of immune checkpoint genes, including CTLA4, PDCD1, and others, was significantly higher than in our CRGPI-high group. Since malignancies with overexpression of immunosuppressive checkpoints are more susceptible to ICIs 46, this finding supports the hypothesis that ICIs are more effective in the CRGPI- low group. Moreover, according to our IPS results, the CRGPI-low group had a higher IPS. Therefore, the hypothesis of this investigation is that immunotherapy may benefit CRGPI-low patients. Moreover, TMB was recently evaluated in prospective clinical trials as a potential biomarker for predicting response to ICI therapy in solid tumors, including EC 33; a high TMB level was significantly associated with a more effective response to ICI and enhanced survival 43. High TMB is associated with a better prognosis for EC patients, which is consistent with the literature, suggesting that TMB may help explain why CRGPI is able to affect the immunotherapy prognosis of EC patients, although the complex mechanisms involved require further study. Mismatch repair deficiency/high microsatellite instability (dMMR/MSI-H) tumors treated with ICB show a durable response and sustained survival benefit, and the combination of ICB therapies could further enhance patient outcomes 43. 25-30% of primary endometrial malignancies are MSI-H, whereas 13-30% of endometrial cancer recurrences are MSI-H or dMMR 34. In our study, MSI-H patients with a lower CRGPI score had a more favorable prognosis, indicating that they also responded more favorably to immunotherapy. The mechanism by which tumors with high MSI and TMB respond better to PD-1 inhibitor therapy may be that more mutated genes produce more tumor antigens, making the tumor more immunogenic and thus resulting in greater lymphocyte infiltration, and that hypermutated tumors may contain more tumor-specific neoantigens and a greater number of tumor-infiltrating lymphocytes (TILs) 42, 45, 47.

Understanding the condition of the TME can facilitate the discovery of novel therapeutics for ECs or the modification of the TME to improve immunotherapy's efficacy. TME is characterized by hypoxia, acidity, and malnutrition, which result from rapid tumor cell proliferation and inadequate angiogenesis; cancer cells respond to various detrimental tumor microenvironments by reprogramming to maintain cancer cell proliferation and growth 48. In EC, the TME contains a greater number of immune cells and cytokines than in other gynecological cancers 43. All stromal, immune, and ESTIMATE scores were higher in the CRGPI-low group, indicating that CRGPI-low may be more immunogenic; the composition of certain immune cells is also distinct between the two groups. B cells naïve, dendritic cells activated, T cells CD4 memory resting, T cells follicular helper, as well as macrophages M1 were more prevalent in the CRGPI-high subgroup. In addition, the results of the ssGSEA algorithm indicated that patients in the CRGPI-low group have a higher level of immune activity. Numerous studies have shown that a concentrated infiltration of T cells, especially cytotoxic CD8 T cells, indicates a favorable prognosis 49, 50. CD8+ counts are an independent factor associated with a favorable prognosis in patients with high-grade EC 51. High dendritic cell density, tumor-associated B lymphocytes, and macrophages were associated with a poor prognosis 52. These cells are associated with a poor prognosis in breast, bladder, ovarian, and prostate cancers. In contrast, a large density of M1 macrophages may indicate a favorable prognosis for ovarian or gastric cancer patients 49, 53. Our findings correspond to these conclusions. The CRGPI-high group had more immunosuppressive cells and signals, whereas the CRGPI-low group had greater immune cell infiltration, higher TMB and MSI-H, and greater immune checkpoint gene expression, indicating that the CRGPI-low group was immune-active and more responsive to ICIs. In conjunction with other molecules and immune subtypes, CRGPI clustering could be used to identify distinct EC molecular and immune subtypes. ICI therapy benefits CRGPI-low patients more than CRGPI-high patients because CRGPI-low patients may have a stronger immune response to tumor initiation. CRGPI-based distinctions in the TME may reflect distinct immune benefits of ICI therapy (anti-PD1 and anti-CTLA4) as identified by IPS. The low CRGPI group had higher IPS scores, indicating increased immunotherapy efficacy.

To further validate the prognostic value and immunotherapeutic benefit prediction of the CRGPI, a survival analysis was undertaken on different immune-therapeutic cohorts receiving anti-PD-L1/PD-1 therapy. We discovered that the CRGPI was able to differentiate various outcomes among patients treated with anti-PD-L1/PD-1 therapies in three independent external cohorts, with an improved prognosis in the CRGPI low group, further validating our CRGPI's strong predictive ability in ICI therapy. Moreover, CRGPI is an excellent predictor of immunotherapeutic response; individuals with a lower CRGPI may have a better immune-therapeutic response, especially to PD-L1 therapy.

Certain biomarkers, including tumor immune dysfunction and exclusion (TIDE) 54 and TIS 23, have been reported to predict patient response to immunotherapy. Due to the fact that ECs are more immunogenic and nearly all TCGA patients had a responder phenotype in the TIDE score, which does not accurately reflect the situation, the TIDE score was not validated in this investigation. As a clinical analysis, TIS provides quantitative and qualitative information about the TME, which is comprised of the expression of 18 genes, such as genes reflecting sustained adaptive Th1 and cytotoxic CD8 T cell responses, and TIS has demonstrated promising results in predicting response to anti-PD-1/PD-L agents. TIS is preoccupied with the function and condition of T cells, which does not fully convey the complexity of TME participation in response to immunotherapy. In addition, TIS focuses on patient response to immunotherapy rather than patient survival time, as well as whether a positive prognosis for patient survival after receiving therapy can be predicted, which appears essential when selecting treatments. Moreover, the CRGPI consists of only six genes, making it much simpler to identify than TIS or other indicators (TMB, MSI, etc.), which has significant health economic benefits.

Despite this, there are several drawbacks to this research. First, this study only considered genes associated with cuproptosis and disregarded other immune-related biomarkers. Second, the number of immune cells varies greatly between individuals, and it is challenging to ascertain whether gene expression levels are primarily dependent on the type of immune cell. Furthermore, this research is based on online data, and large clinical sample studies are still necessary to validate the predictive value of the CRGPI model.

CRGPI is a promising cuproptosis-related prognostic biomarker in EC, in conclusion. CRGPI grouping aids in differentiating immune and molecular signatures, predicts patient prognosis, and may be a potential prognostic indicator for immunotherapy; however, more research is necessary to elucidate this.

## Supplementary Material

Supplementary figures.

Supplementary tables.

## Figures and Tables

**Table 1:** Cell count for each sample in GSE154763.

GSM	Patient	Raw-count	Clean-count	Percent %
GSM4679335	P20190312	189	189	100.00
GSM4679336	P20181122	609	606	99.51
GSM4679337	P20181211	52	50	96.15
GSM4679338	P20190213	334	329	98.50
GSM4679339	P20190305	513	512	99.81
GSM4679340	P20190625	254	244	96.06
GSM4679341	P20190717	349	345	98.85
GSM4679342	P20190910	2543	2535	99.69
GSM4679343	P20190911	3965	3957	99.80

**Table 2:** Multivariate Cox regression analysis of prognostic CRGs associated with overall survival in EC patients.

gene	coef	HR	HR.95L	HR.95H	p value
CDKN2A	0.00807812	1.00811083	0.99919657	1.01710462	0.07465141
PDHA1	0.02499727	1.02531232	1.00640327	1.04457666	0.00848757
GLS	0.04456927	1.0455774	1.00835584	1.08417293	0.0159572
DBT	0.3117633	1.36583136	1.05008194	1.77652356	0.02010988
SLC31A1	-0.0691128	0.93322139	0.86122545	1.01123599	0.0915653

## Abbreviations

EC: endometrial cancer
CRGs: cuproptosis-related genes
CRGPI: cuproptosis-related gene prognostic index
scRNA: single-cell RNA
ICI: immune checkpoint inhibitor
PD1: programmed death 1
PD-L1: programmed death ligand 1
CTLA4: CTL associated protein 4
IPS: immunophenoscore
TIS: tumor inflammation signature
