# Supplementary material for: Coordination of Single-cell and Bulk RNA Sequencing to Construct a Cuproptosis-related Gene Prognostic Index for Endometrial Cancer Prognosis, Immune Microenvironment Infiltration, and Immunotherapy Treatment Options
Source: J Cancer. 2023 Sep 18;14(16):3078–98. doi: 10.7150/jca.86325 (PMC10583581; doi:10.7150/jca.86325)
Supplement: Supplementary file 1 — Supplementary figures. [file jcav14p3078s1.pdf]

Supplementary figures:

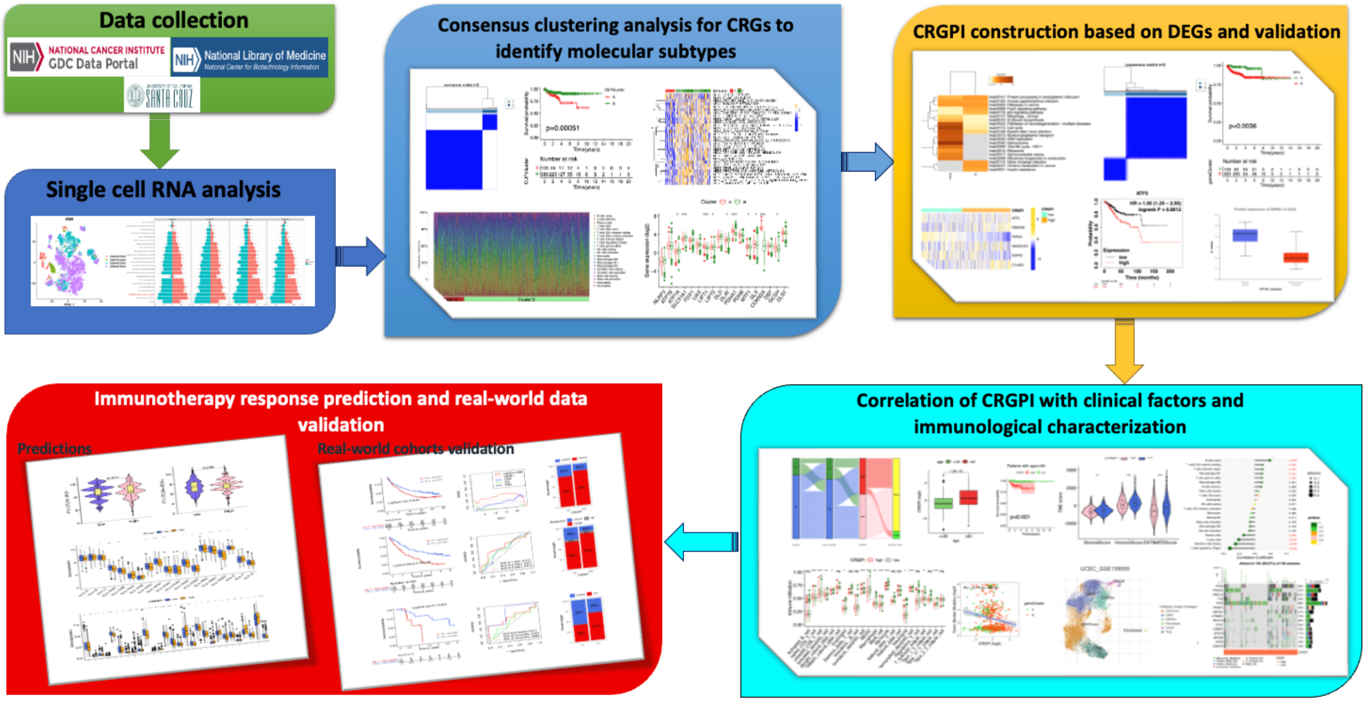

Figure S1. Flowchart of the entire analytical process in the study.

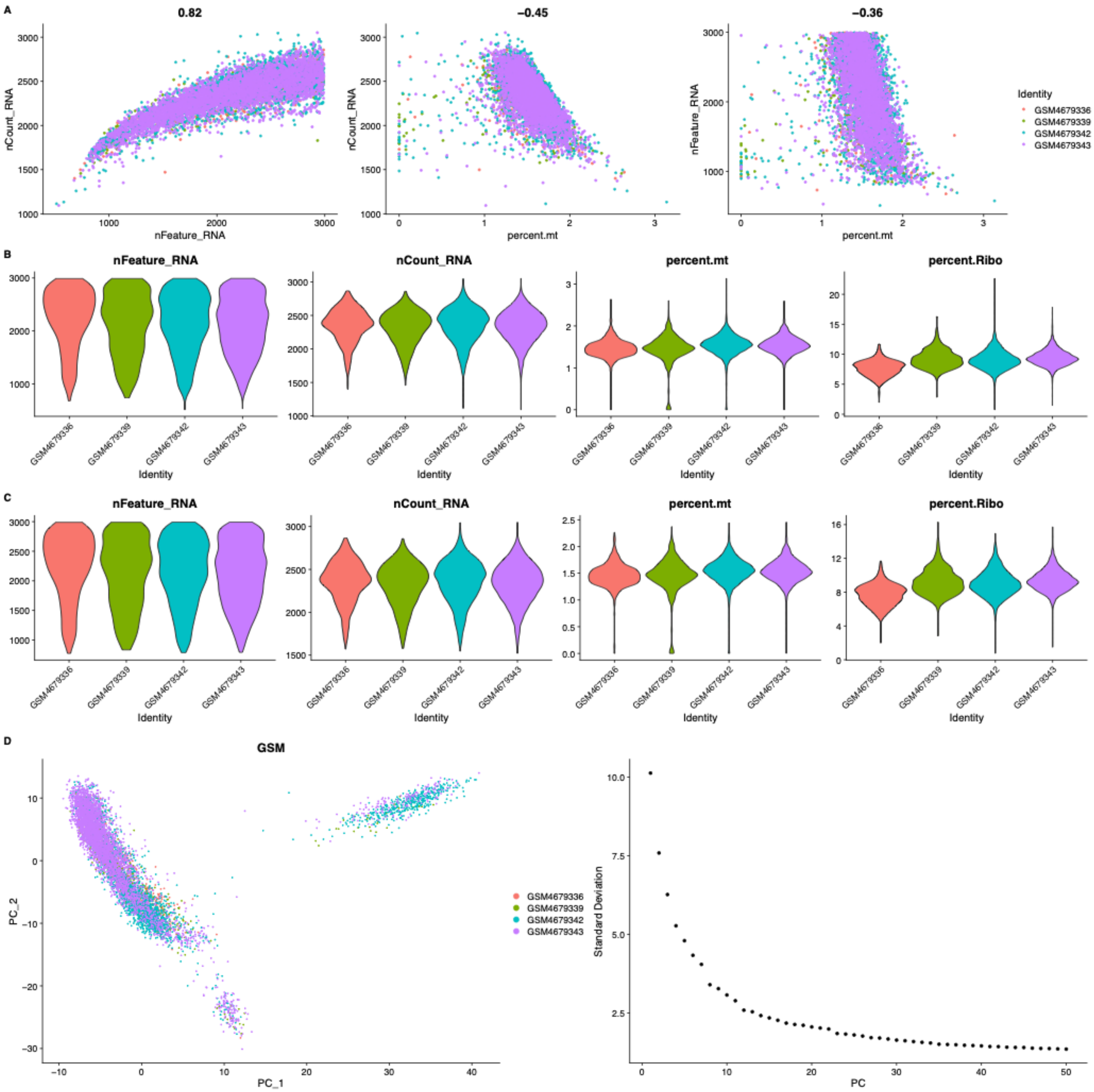

Figure S2. scRNA-seq analysis of four EC samples.

(A) The relationship between mitochondrial gene and the number of UMI/mRNA, as well as the correlation between UMI and mRNA.

(B, C) Quality control, which includes the number of unique genes and total molecules as well as the proportion of sequences that map to the mitochondrial genome.

(D) Based on scRNA-seq data, the PCA confirms the top 50 PCs.

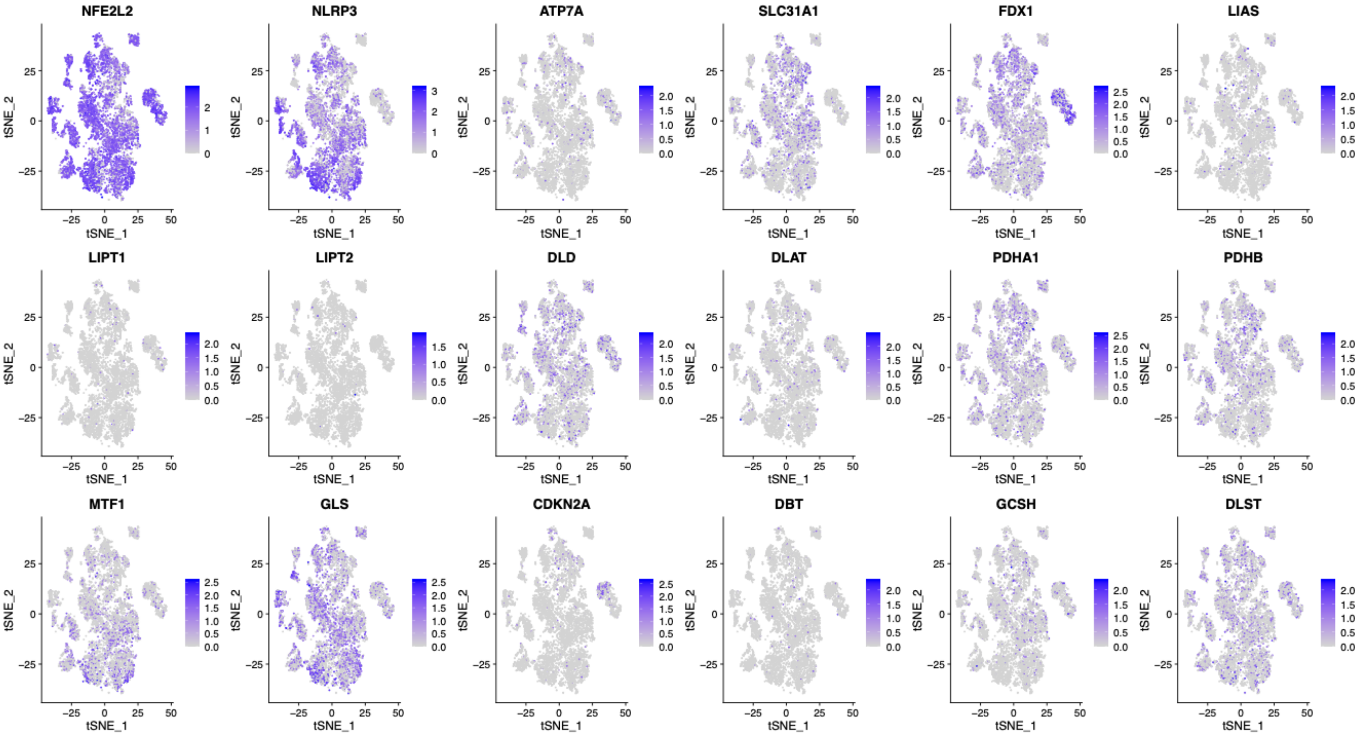

Figure S3. Single-cell subpopulation distributions of CRG-related genes

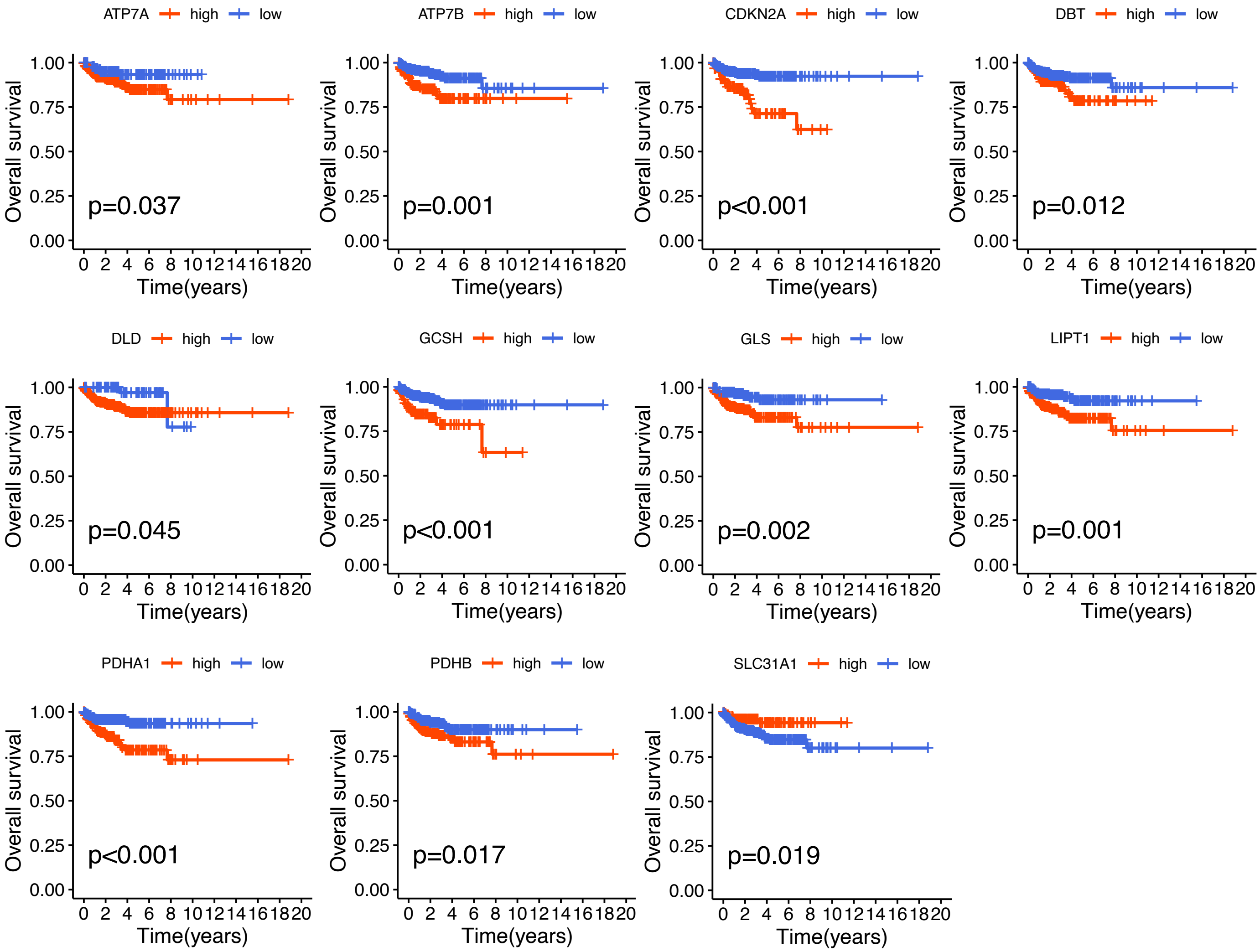

Figure S4. The Kaplan-Meier plots of 11 prognostic CRGs in EC.

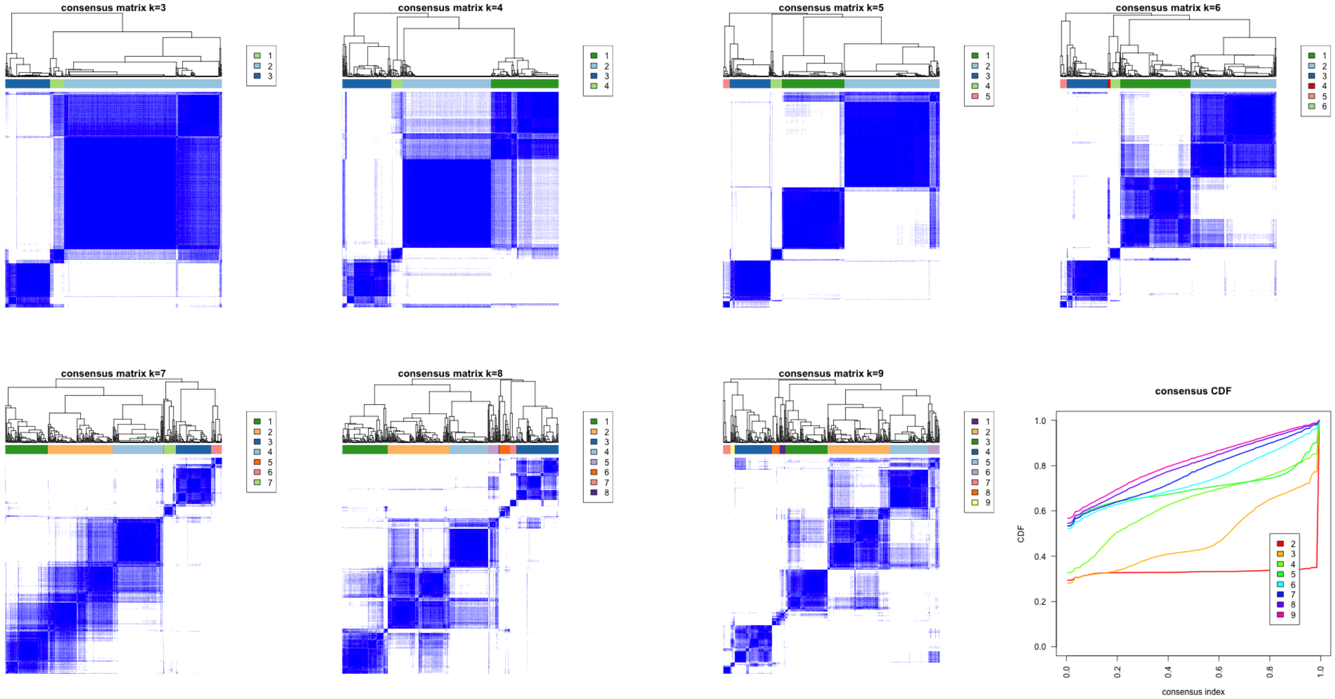

Figure S5. Unsupervised clustering of cuproptosis-related genes and Consensus matrix heatmaps for k = 3-9, as well as the consensus cumulative distribution function (CDF) curve for 2-9 curves.

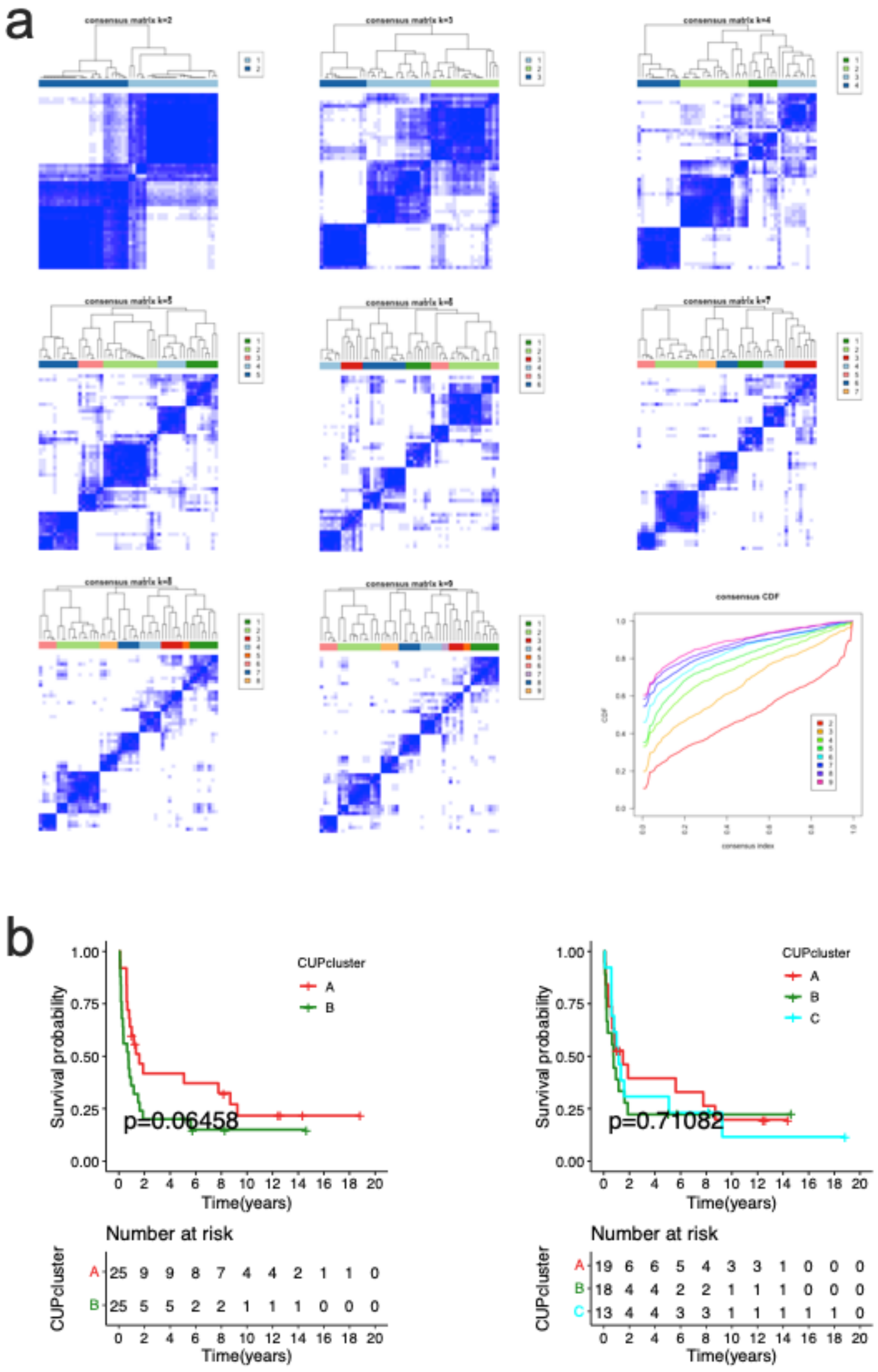

Figure S6. External validation for unsupervised clustering of cuproptosis-related genes in GSE119041.

a, unsupervised clustering of cuproptosis-related genes in GSE119041, showing the consensus matrix heatmaps for k = 2-9, as well as the consensus cumulative distribution function (CDF) curve for 2-9 curves

b, Kaplan–Meier curves for overall survival of 50 undifferentiated uterine sarcomas samples (GSE119041) with two and three cuproptosis subtypes, the differences were observed. When k=2, the KM prognosis analysis suggested that the subtyping had a more different prognosis, which was significantly better than the other subtypes (log-rank test, p=0.06458 for two clusters, 0.071082 for three clusters).

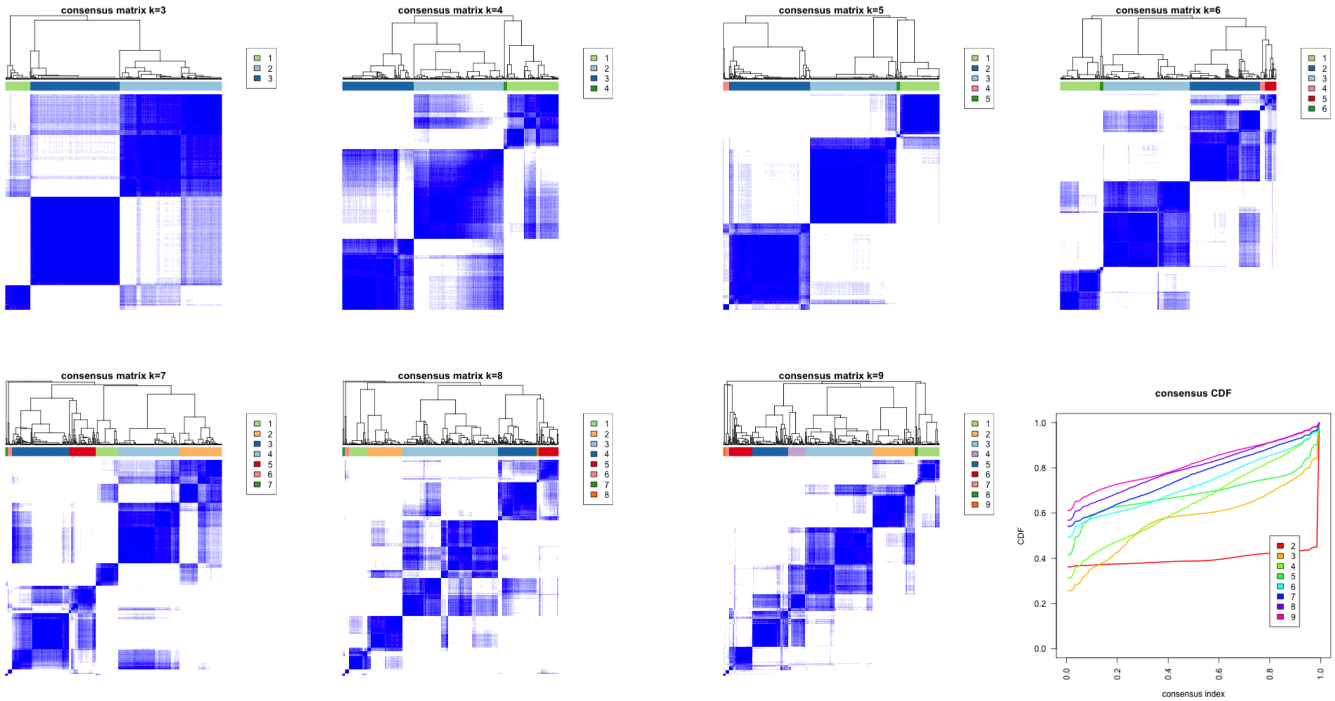

Figure S7. Unsupervised clustering of prognostic DEGs and Consensus matrix heatmaps for k = 3-9, as well as the consensus cumulative distribution function (CDF).

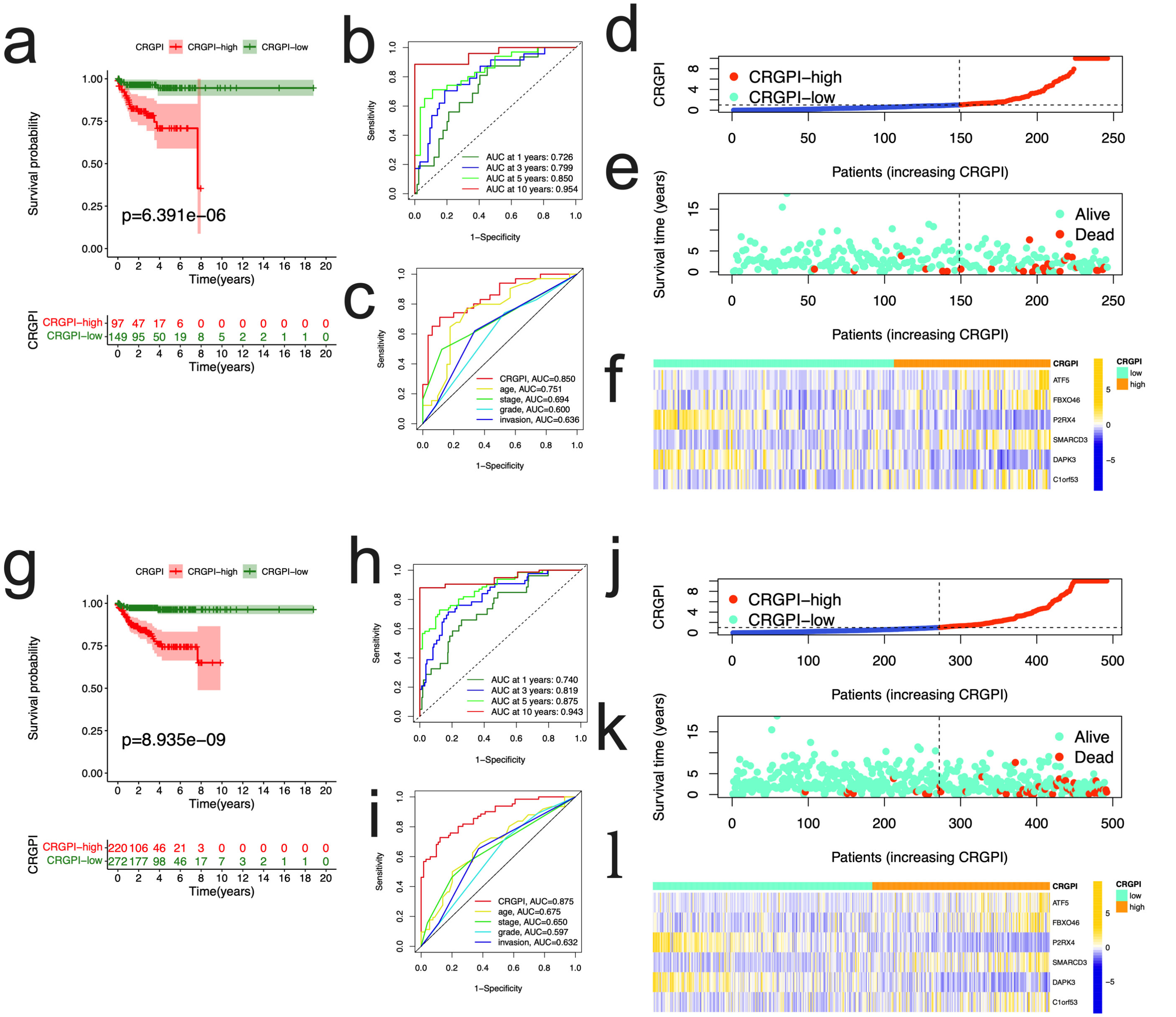

Figure S8. Internal validation of the CRGPI in testing cohorts and overall cohorts.

Kaplan–Meier curves for overall survival (OS) of the patients from the CRGPI-high and -low groups in the testing cohorts (a) and overall cohorts (g);

ROC curves and CRGPI AUC values showed 1-, 3-, 5-, and 10-year predictions in the testing cohorts (b) and overall cohorts (h);

ROC curves of CRGPI and clinicopathological factors (age, grade, stage, and myometrial invasion) for 5-year AUC in the testing cohorts (c) and overall cohorts (i);

Risk score distribution plot showed the distribution of CRGPI-high and -low groups in the testing cohorts (d) and overall cohorts (j);

Scatter plot showed the correlation between the survival status and CRGPI in the testing cohorts (e) and overall cohorts (k).

Heatmap plots showed the expression profile of seven genes in CRGPI-high and -low groups in the testing cohorts (f) and overall cohorts (l).

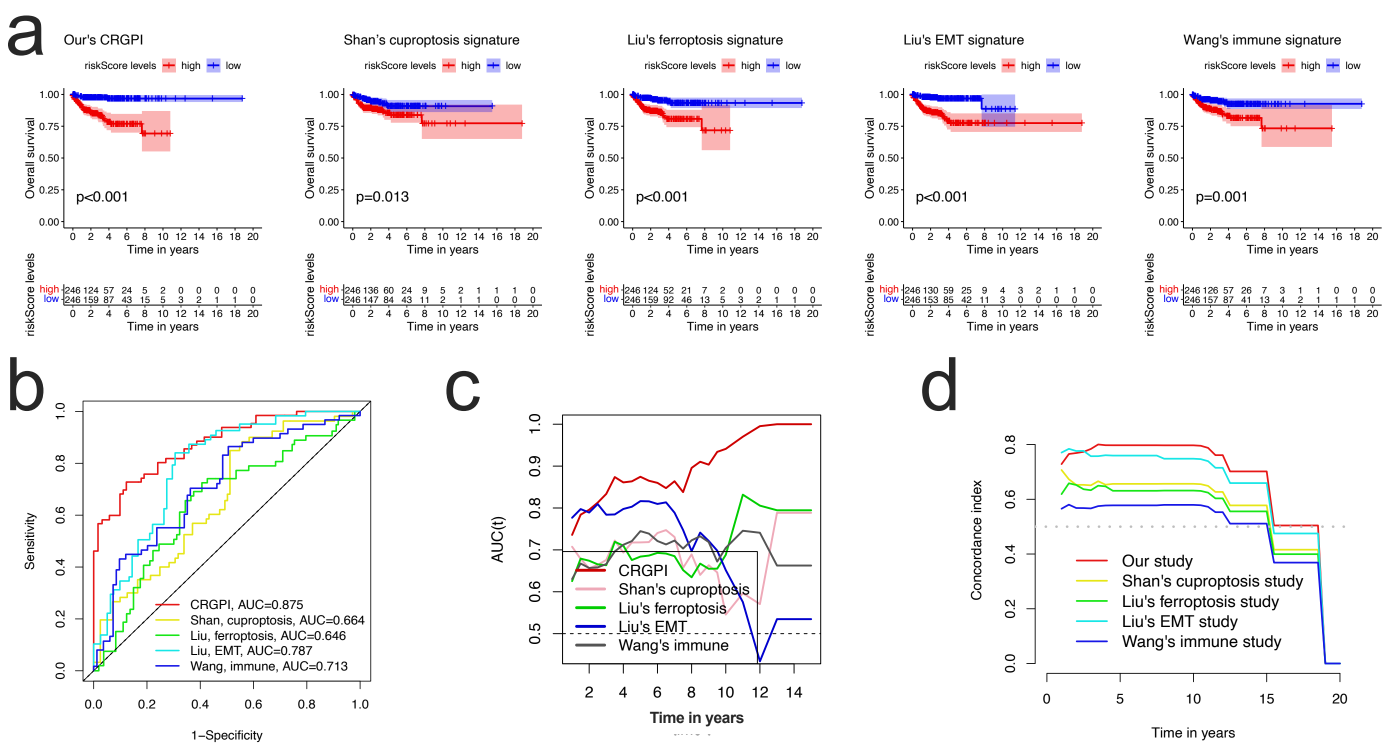

Figure S9. Comparison of our model with other four previously established models

a. Survival curves of our model and four previous published gene-signatures.

b. ROC curves of our model and four previous published gene-signatures.

c. Time-dependent ROC curves of our model and four previous published gene-signature.

d. C-index plot of our model and four previous published gene-signature for 0-20 years in TCGA EC database.

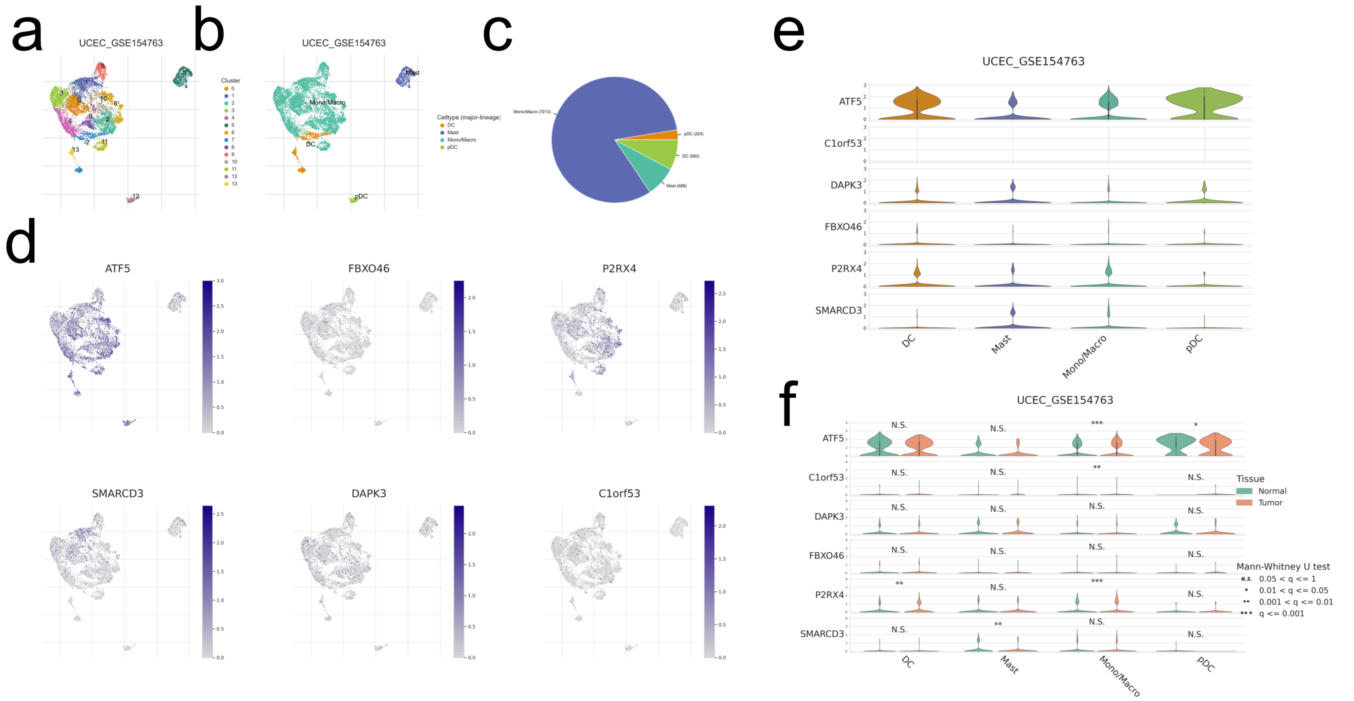

Figure S10. CRGPI gene related cell type distribution using scRNA seq database

(a-c) The cell types and their distribution in UCEC GSE154763 dataset; d. distribution of CRGPI six genes in different cells in UCEC GSE154763 dataset; e. Distribution of six CRGPI gene's expression in different cell types using violin plot; f. Distribution of six CRGPI gene's expression in different cell types of normal endometrial and tumor cells.

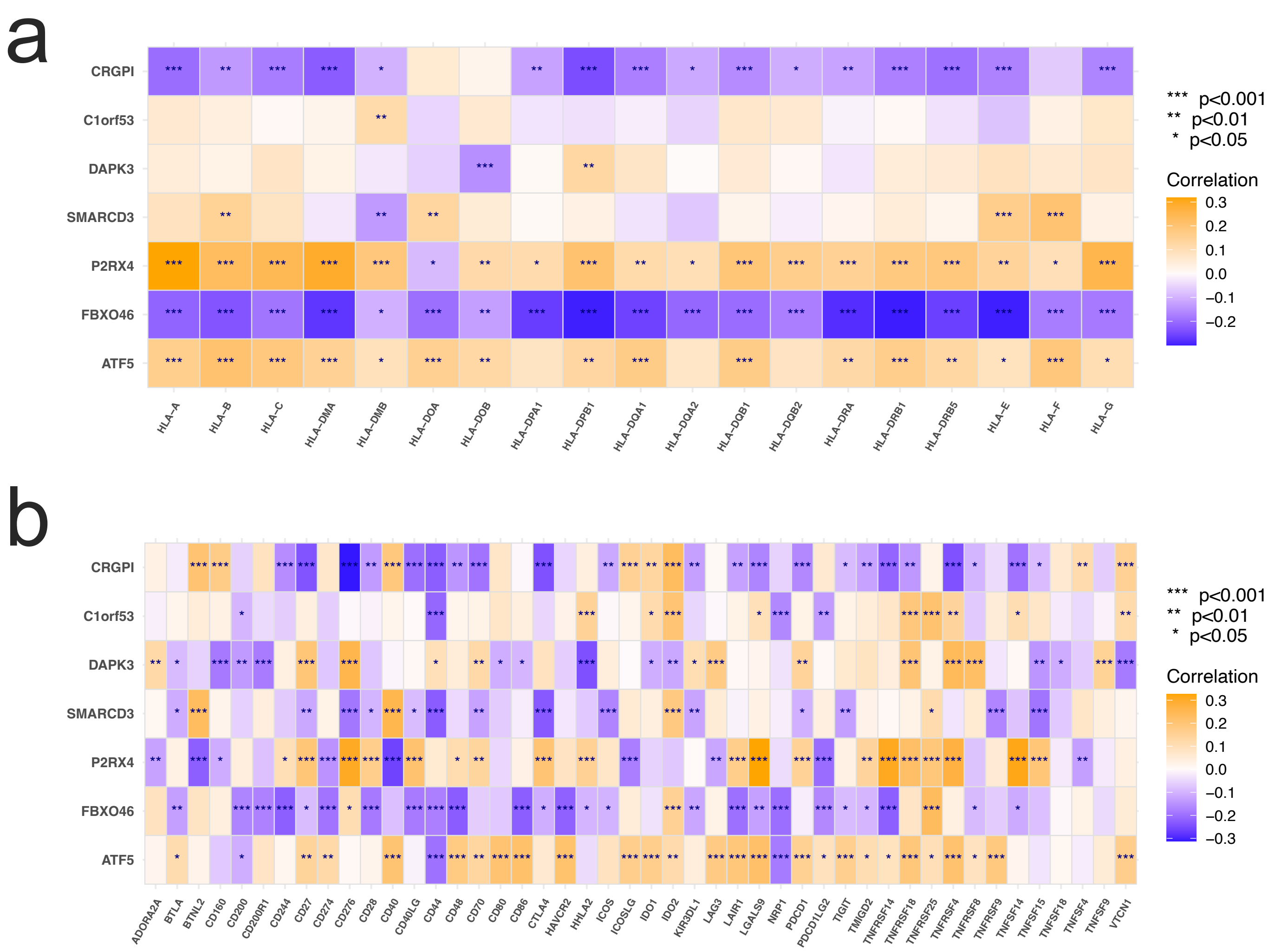

Figure S11. The correlation of six CRGPI genes with HLA family genes and check-point genes

a. the correlation of six CRGPI genes with HLA family genes;

b. the correlation of six CRGPI genes with check-point genes.

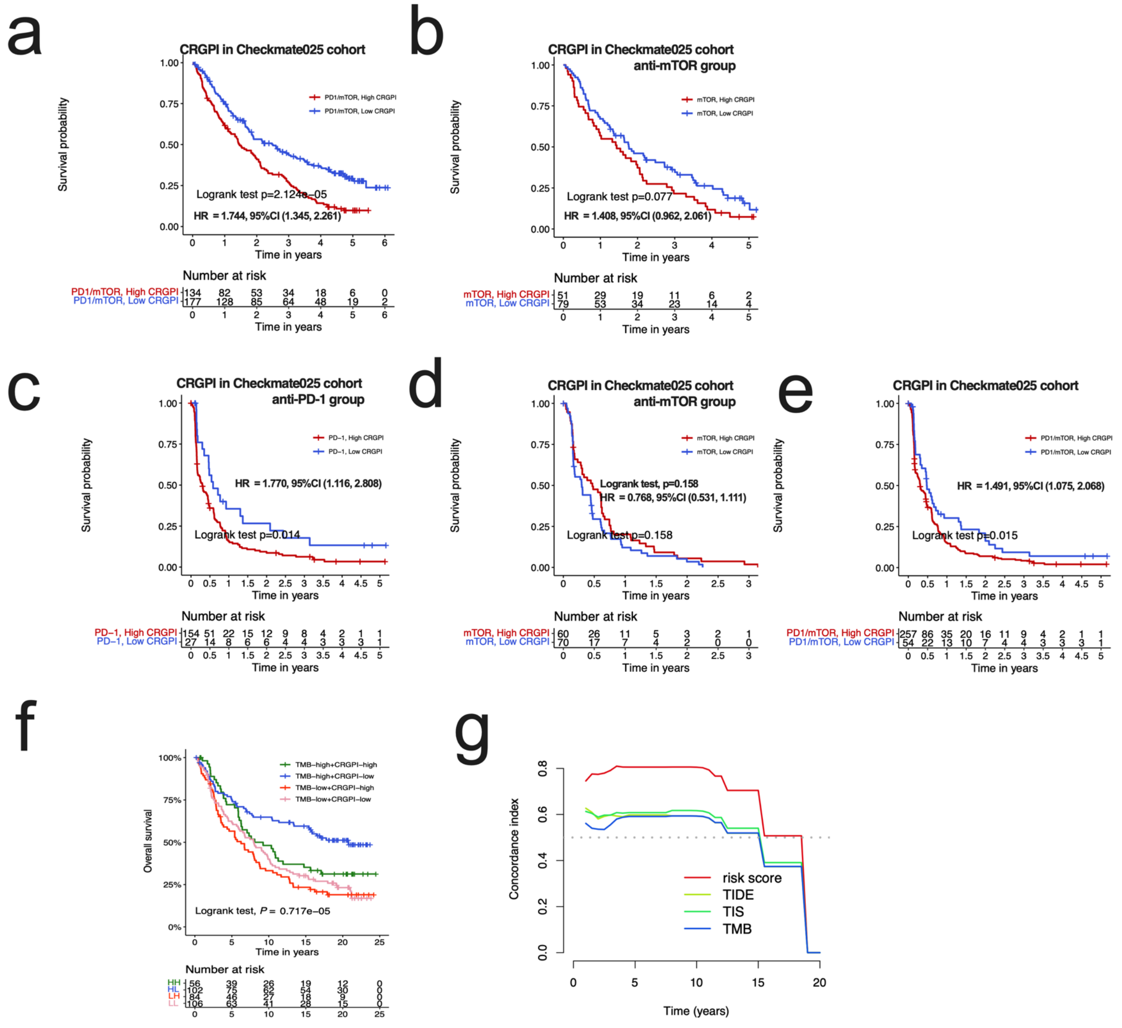

Figure S12. Comparison of CRGPI with other indications for prognosis prediction and evaluation of CRGPI in tumor immunotherapy response in different cohorts.

Kaplan–Meier survival analysis of OS for the CRGPI subgroups in CheckMate025 total cohort (a) and anti-mTOR sub-cohort (b); Kaplan–Meier survival analysis of progression-free survival for the CRGPI subgroups in CheckMate025 anti-PD-1 sub-cohort (c), anti-mTOR sub-cohort (d), and total-cohort (e); Kaplan–Meier curves of OS of patients treated with anti-PD-L1 stratified by both TMB and CRGPI in IMvigor210 cohort (f); The comparison of concordance index for CRGPI, TIDE, TIS, and TMB in 0-20 years in EC patients of TCGA cohort (g).

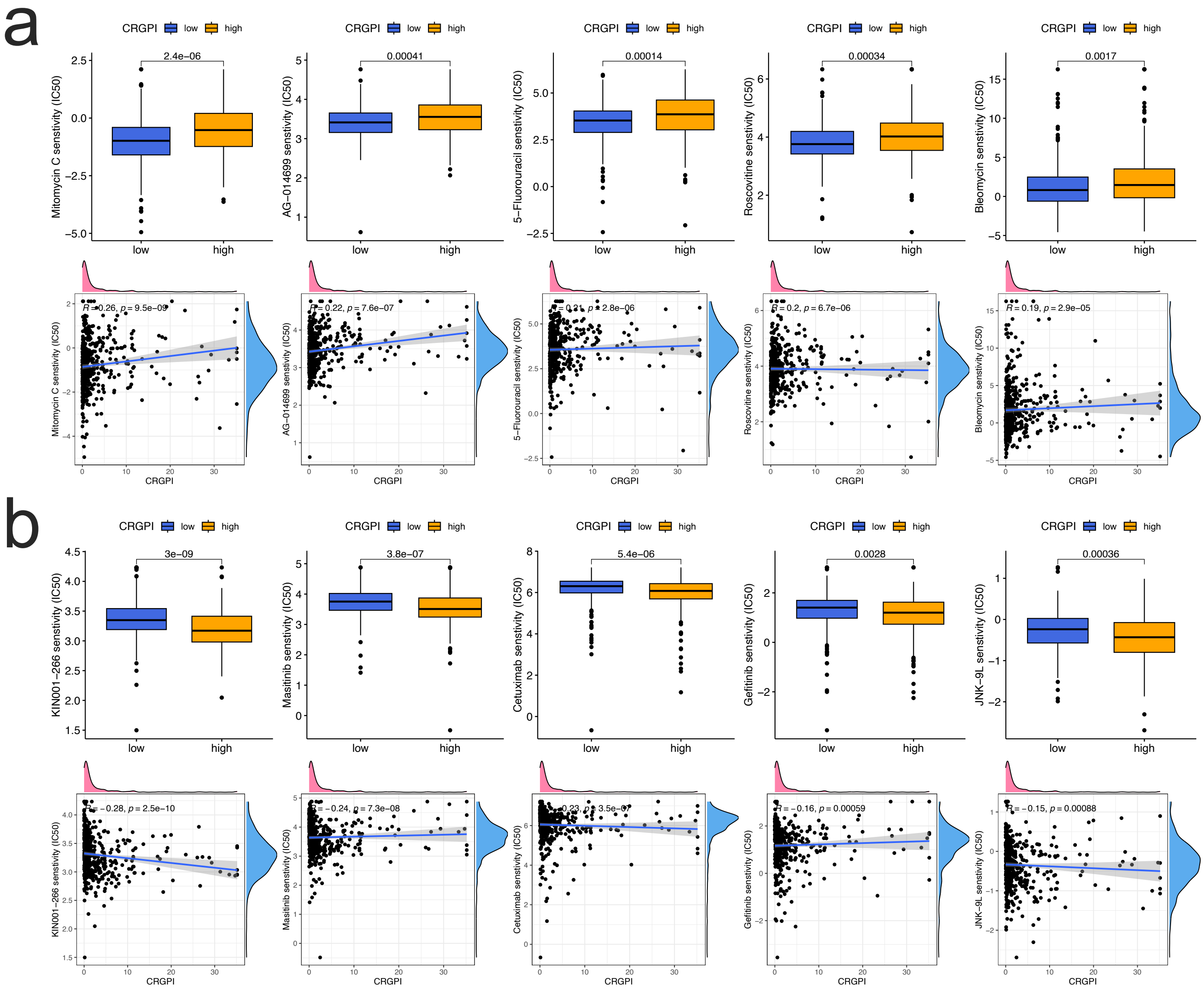

Figure S13. Association of the CRGPI with drug prediction.

a. Differences in drug sensitivity between the CRGPI-high and CRGPI-low groups based on IC50 values of Mitomycin C, PARP inhibitor (ag-014699), 5-Fluorouracil, CDK inhibitor (Roscovitine), Bleomycin , the corresponding correlations between CRGPI and IC50 values were showing below.

b. Differences in drug sensitivity between the CRGPI-high and CRGPI-low groups based on IC50 values of p38 MAPK inhibitor (KIN001-266), tyrosine kinase inhibitor (Masitinib), EGFR antibody (Cetuximab, Gefitinib), JNK inhibitor (JNK-9L), the corresponding correlations between CRGPI and IC50 values were showing below.
